# Black flies (Diptera: Simuliidae) of Turkish Thrace, with a new record for Turkey

**DOI:** 10.3897/BDJ.3.e4834

**Published:** 2015-04-22

**Authors:** Ümit Davut Şirin, Hakan Çalışkan, Yalçın Şahin

**Affiliations:** ‡ Eskişehir Osmangazi University, Faculty of Science and Art, Biology Department, Eskişehir, Turkey

**Keywords:** Black flies, Thrace, Turkey, biodiversity

## Abstract

**Background:**

This paper includes 2742 specimens of 18 species of black flies (Diptera: Simuliidae) collected from 132 lotic sites in Turkish Thrace, the European part of Turkey, in the early summer of 2002 and 2003 and the spring of 2005 and 2006.

**New information:**

All species are recorded from this region for the first time, and *Metacnephia nigra* (Rubtsov, 1940) is a new record for Turkey. Distributional and taxonomical remarks are given for each species.

## Introduction

Black flies (Simuliidae) are blood-sucking flies, considered the third most medically and veterinary important arthropod group (Adler et al. 2004). A total of 2,177 living, formally described species of black flies are currently recognized as valid, with new species continuing to be discovered (Adler and Crosskey 2015) The immature stages play an important ecological role in streams and rivers where they often dominate the benthos (Crosskey 1990).

TheTurkish simuliid fauna is not well known, although investigations have increased in the past 15 years. The first report of the family in Anatolia was by Austen (1925) who described *Simulium pulchripes* from the Asiatic part of Çanakkale Province. After 50 years, a second species, *Simulium caucasicum* (as *Odagmia ornata caucasica*), was recorded by Jedlićka (1975) from Çanakkale and Afyon Provinces. In the second edition of *Limnofauna Europea* (Zwick 1978), three species, *Simulium bezzii*, *S. balcanicum* and *S. pseudequinum* (as *mediterraneum*), were recognized from Anatolia (“Kl. Asien”) (Crosskey and Zwick 2007). Kazancı and Clergue-Gazeau (1990) published a detailed study of Turkish black flies and recorded 21 species from Anatolia. In a later paper, they added ecological data and species records (Clergue-Gazeau and Kazancı 1992). An outbreak of *S. bezzii* in eastern Anatolia in Erzurum Province was reported by Özbek et al. (1995). Balık et al. (2002) recorded *S. angustitarse* from Dikili District of İzmir Province. Ümit Şirin completed his doctoral thesis on black flies in the Upper Sakarya River basin in northwestern Anatolia and published eight new records for Turkey (Sirin and Sahin 2005). An account of the Turkish Simuliidae, with nine new records and a checklist of 40 species, was published by Crosskey and Zwick (2007). A second checklist included 63 species from previous publications and new records was published by Kazancı and Ertunç (2008). Çağlar and İpekdal (2009), however, indicated that 45 species were known from Turkey. The most recent record from Turkey was reported by Kalafat and Şirin (2011). The latest version of the “Inventory of World Blackflies” lists 51 species for Turkey (Adler and Crosskey 2015).

Turkish Thrace, the European part of the country, is divided from Asia Minor by the Turkish straits (Bosporus and Dardanelles). It is bounded by Bulgaria to the north, Greece to the west, the Black Sea to the northeast and Marmara and the Aegean Sea to the south. The largest mountain range is Yıldız, about 1031 m above sea level. The Meriç and Tunca Rivers from Bulgaria and the Arda River from Greece cross the region and empty into the Aegean Sea. Wetlands in the region provide resting and staging areas for birds that migrate between Europe and Asia. Agriculture uses modern technology in the vast lowlands and productive plains of the region. Pollution from agriculture, industry and excessive population growth affects a large portion of the freshwater ecosystems (Erdoğan and Güher 2012).

Most faunal records of Turkish black flies are from Anatolia, the Asian part of the country, and there is only one previous record [*Simulium ornatum* Meigen s.l. from İstanbul; (Zwick 2007)] dealing with black flies in the European part of Turkey. We report 18 species of black flies from Turkish Thrace, as well as one of from Turkey, for the first time.

## Materials and methods

**Figure 1.**
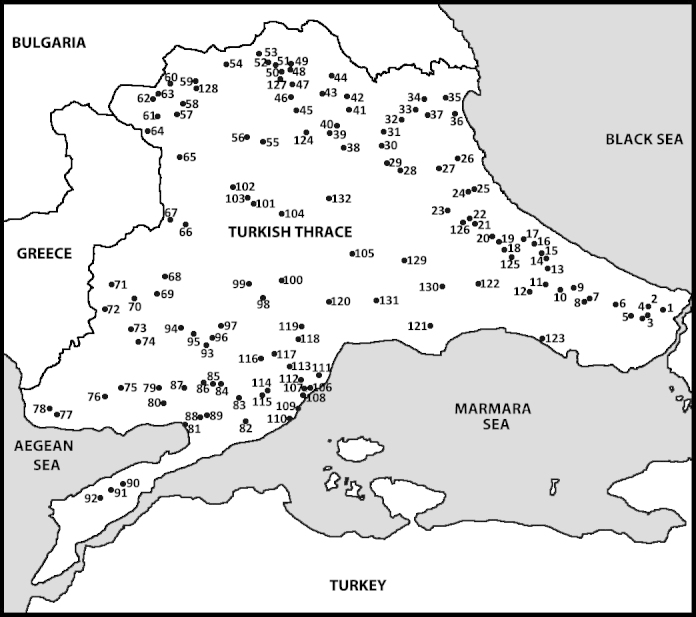
Map of collecting sites for black flies in Turkish Thrace. Suppl. material 1.

Study material was based on aquatic stages and reared adults from 132 rivers, streams and springs in the region (Fig. 1). The material included 2742 specimens (1168 larvae, 1369 pupae, 204 pharate adults and 47 reared adults) and was deposited in the LAB).

Larvae and pupae were collected into 80% ethanol, and reared flies with their pupal exuviae also were fixed in ethanol. For each species, the numbers of larvae, pupae and adults collected at a sampling site on a given date are recorded in the examined material below. Material was studied according to methods described by Bass (1998), with the aid of a stereomicroscope (Leica MZ 16).

Identifications were made using the keys and descriptions by Rubtsov (1956), Knoz (1965), Terteryan (1968), Crosskey (2002), Bass (1998), Crosskey and Malicky (2001), Yankovsky (1995), Crosskey and Zwick (2007) and Jedlićka et al. (2004). The nomenclature follows that of Adler and Crosskey (2015).

## Taxon treatments

### Prosimulium rachiliense

Djafarov, 1954

#### Materials

- **Type status:** Other material. **Occurrence:** lifeStage: 43 pupae, 6 larvae; **Taxon:** scientificName: *Prosimulium rachiliense* Djafarov,1954; kingdom: Animalia; phylum: Arthropoda; class: Insecta; order: Diptera; family: Simuliidae; genus: Prosimulium; scientificNameAuthorship: Djafarov,1954; **Location:** continent: Europe; country: Turkey; stateProvince: Kırklareli; county: Merkez; locationRemarks: 46; verbatimLatitude: 41°49'48.43"N; verbatimLongitude: 27°10'42.01"E; **Identification:** identificationID: esoguent-th-ıd-1; **Event:** eventDate: 04/23/2005
- **Type status:** Other material. **Occurrence:** lifeStage: 33 pupae, 6 larvae; **Taxon:** scientificName: *Prosimulium rachiliense* Djafarov,1954; kingdom: Animalia; phylum: Arthropoda; class: Insecta; order: Diptera; family: Simuliidae; genus: Prosimulium; scientificNameAuthorship: Djafarov,1954; **Location:** continent: Europe; country: Turkey; stateProvince: Kırklareli; county: Kofçaz; locationRemarks: 49; verbatimLatitude: 41°55'33.95"N; verbatimLongitude: 27°9'48.15"E; **Identification:** identificationID: esoguent-th-ıd-2; **Event:** eventDate: 04/23/2005
- **Type status:** Other material. **Occurrence:** lifeStage: 6 pupae, 1 larvae; **Taxon:** scientificName: *Prosimulium rachiliense* Djafarov,1954; kingdom: Animalia; phylum: Arthropoda; class: Insecta; order: Diptera; family: Simuliidae; genus: Prosimulium; scientificNameAuthorship: Djafarov,1954; **Location:** continent: Europe; country: Turkey; stateProvince: Kırklareli; county: Kofçaz; locationRemarks: 50; verbatimLatitude: 41°56'53.66"N; verbatimLongitude: 27°9'42.23"E; **Identification:** identificationID: esoguent-th-ıd-3; **Event:** eventDate: 04/23/2005
- **Type status:** Other material. **Occurrence:** lifeStage: 11 pupae, 6 larvae; **Taxon:** scientificName: *Prosimulium rachiliense* Djafarov,1954; kingdom: Animalia; phylum: Arthropoda; class: Insecta; order: Diptera; family: Simuliidae; genus: Prosimulium; scientificNameAuthorship: Djafarov,1954; **Location:** continent: Europe; country: Turkey; stateProvince: Kırklareli; county: Kofçaz; locationRemarks: 51; verbatimLatitude: 41°56'22.44"N; verbatimLongitude: 27°5'33.09"E; **Identification:** identificationID: esoguent-th-ıd-4; **Event:** eventDate: 04/23/2005
- **Type status:** Other material. **Occurrence:** lifeStage: 12 pupae, 1 larvae; **Taxon:** scientificName: *Prosimulium rachiliense* Djafarov,1954; kingdom: Animalia; phylum: Arthropoda; class: Insecta; order: Diptera; family: Simuliidae; genus: Prosimulium; scientificNameAuthorship: Djafarov,1954; **Location:** continent: Europe; country: Turkey; stateProvince: Edirne; county: Lalapaşa; locationRemarks: 53; verbatimLatitude: 41°58'26.47"N; verbatimLongitude: 27°0'28.78"E; **Identification:** identificationID: esoguent-th-ıd-5; **Event:** eventDate: 04/23/2005
- **Type status:** Other material. **Occurrence:** lifeStage: 4 pupae, 1 larvae; **Taxon:** scientificName: *Prosimulium rachiliense* Djafarov,1954; kingdom: Animalia; phylum: Arthropoda; class: Insecta; order: Diptera; family: Simuliidae; genus: Prosimulium; scientificNameAuthorship: Djafarov,1954; **Location:** continent: Europe; country: Turkey; stateProvince: Tekirdağ; county: Şarköy; locationRemarks: 82; verbatimLatitude: 40°41'28.95"N; verbatimLongitude: 27°5'45.01"E; **Identification:** identificationID: esoguent-th-ıd-6; **Event:** eventDate: 04/24/2005
- **Type status:** Other material. **Occurrence:** lifeStage: 4 pupae, 2 larvae; **Taxon:** scientificName: *Prosimulium rachiliense* Djafarov,1954; kingdom: Animalia; phylum: Arthropoda; class: Insecta; order: Diptera; family: Simuliidae; genus: Prosimulium; scientificNameAuthorship: Djafarov,1954; **Location:** continent: Europe; country: Turkey; stateProvince: Tekirdağ; county: Şarköy; locationRemarks: 83; verbatimLatitude: 40°46'21.52"N; verbatimLongitude: 27°3'32.09"E; **Identification:** identificationID: esoguent-th-ıd-7; **Event:** eventDate: 04/24/2005
- **Type status:** Other material. **Occurrence:** lifeStage: 3 larvae; **Taxon:** scientificName: *Prosimulium rachiliense* Djafarov,1954; kingdom: Animalia; phylum: Arthropoda; class: Insecta; order: Diptera; family: Simuliidae; genus: Prosimulium; scientificNameAuthorship: Djafarov,1954; **Location:** continent: Europe; country: Turkey; stateProvince: Kırklareli; county: Saray; locationRemarks: 126; verbatimLatitude: 41°26'20.97"N; verbatimLongitude: 28°4'43.83"E; **Identification:** identificationID: esoguent-th-ıd-8; **Event:** eventDate: 04/22/2005
- **Type status:** Other material. **Occurrence:** lifeStage: 14 pupae, 9 larvae; **Taxon:** scientificName: *Prosimulium rachiliense* Djafarov,1954; kingdom: Animalia; phylum: Arthropoda; class: Insecta; order: Diptera; family: Simuliidae; genus: Prosimulium; scientificNameAuthorship: Djafarov,1954; **Location:** continent: Europe; country: Turkey; stateProvince: Kırklareli; county: Kofçaz; locationRemarks: 127; verbatimLatitude: 41°55'2.35"N; verbatimLongitude: 27°7'37.70"E; **Identification:** identificationID: esoguent-th-ıd-9; **Event:** eventDate: 04/23/2005
- **Type status:** Other material. **Occurrence:** lifeStage: 38 pupae, 7 larvae; **Taxon:** scientificName: *Prosimulium rachiliense* Djafarov,1954; kingdom: Animalia; phylum: Arthropoda; class: Insecta; order: Diptera; family: Simuliidae; genus: Prosimulium; scientificNameAuthorship: Djafarov,1954; **Location:** continent: Europe; country: Turkey; stateProvince: Edirne; county: Lalapaşa; locationRemarks: 128; verbatimLatitude: 41°50'8.01"N; verbatimLongitude: 26°43'49.22"E; **Identification:** identificationID: esoguent-th-ıd-10; **Event:** eventDate: 04/23/2005

#### Notes

This taxon is distributed mainly in Caucasia, Transcaucasia and the Balkan countries including the island of Rhodes, and its occurrence in Turkey has been known previously (Crosskey and Zwick 2007). The first record from Anatolia was by Kazancı and Clergue-Gazeau (1990), as *P. pronevitschae*, from three different lotic systems. Crosskey and Howard (2004) synonymized *pronevitschae* with *rachiliense.* Crosskey and Zwick (2007) reported this species from seven sites in northwestern and southeastern Anatolia. We identified our material as *P. rachiliense*, using the illustrations and descriptions by Terteryan (1968) and Crosskey and Malicky (2001). Adler and Şirin (2014) chromosomally showed that populations in western Turkey are a cytoform of *P. rachiliense*, probably different from true *P. rachiliense*. We compared our larvae and pupae with the material in the study of Adler and Şirin (2014)​, and did not find structural differences.

### Metacnephia nigra

(Rubtsov, 1940)

#### Materials

- **Type status:** Other material. **Occurrence:** lifeStage: 1 pupae; **Taxon:** scientificName: *Metacnephia nigra* (Rubtsov, 1940); kingdom: Animalia; phylum: Arthropoda; class: Insecta; order: Diptera; family: Simuliidae; genus: Metacnephia; scientificNameAuthorship: (Rubtsov, 1940); **Location:** continent: Europe; country: Turkey; stateProvince: Kırklareli; county: Demirköy; locationRemarks: 35; verbatimLatitude: 41°52'21.65"N; verbatimLongitude: 27°54'3.96"E; **Identification:** identificationID: esoguent-th-ıd-11; **Event:** eventDate: 04/22/2005
- **Type status:** Other material. **Occurrence:** lifeStage: 12 pupae, 2 larvae; **Taxon:** scientificName: *Metacnephia nigra* (Rubtsov, 1940); kingdom: Animalia; phylum: Arthropoda; class: Insecta; order: Diptera; family: Simuliidae; genus: Metacnephia; scientificNameAuthorship: (Rubtsov, 1940); **Location:** continent: Europe; country: Turkey; stateProvince: Kırklareli; county: İğneada; locationRemarks: 36; verbatimLatitude: 41°49'16.69"N; verbatimLongitude: 27°57'8.53"E; **Identification:** identificationID: esoguent-th-ıd-12; **Event:** eventDate: 04/22/2005
- **Type status:** Other material. **Occurrence:** lifeStage: 2 pupae; **Taxon:** scientificName: *Metacnephia nigra* (Rubtsov, 1940); kingdom: Animalia; phylum: Arthropoda; class: Insecta; order: Diptera; family: Simuliidae; genus: Metacnephia; scientificNameAuthorship: (Rubtsov, 1940); **Location:** continent: Europe; country: Turkey; stateProvince: Kırklareli; county: Merkez; locationRemarks: 46; verbatimLatitude: 41°49'48.43"N; verbatimLongitude: 27°10'42.01"E; **Identification:** identificationID: esoguent-th-ıd-13; **Event:** eventDate: 04/23/2005
- **Type status:** Other material. **Occurrence:** lifeStage: 5 pupae; **Taxon:** scientificName: *Metacnephia nigra* (Rubtsov, 1940); kingdom: Animalia; phylum: Arthropoda; class: Insecta; order: Diptera; family: Simuliidae; genus: Metacnephia; scientificNameAuthorship: (Rubtsov, 1940); **Location:** continent: Europe; country: Turkey; stateProvince: Edirne; county: Lalapaşa; locationRemarks: 53; verbatimLatitude: 41°58'26.47"N; verbatimLongitude: 27°0'28.78"E; **Identification:** identificationID: esoguent-th-ıd-14; **Event:** eventDate: 04/23/2005
- **Type status:** Other material. **Occurrence:** lifeStage: 1 pupae; **Taxon:** scientificName: *Metacnephia nigra* (Rubtsov, 1940); kingdom: Animalia; phylum: Arthropoda; class: Insecta; order: Diptera; family: Simuliidae; genus: Metacnephia; scientificNameAuthorship: (Rubtsov, 1940); **Location:** continent: Europe; country: Turkey; stateProvince: Edirne; county: Lalapaşa; locationRemarks: 128; verbatimLatitude: 41°50'8.01"N; verbatimLongitude: 26°43'49.22"E; **Identification:** identificationID: esoguent-th-ıd-15; **Event:** eventDate: 04/23/2005

#### Notes

We report this species for the first time from Turkey. The taxonomy of the genus *Metacnephia* requires revision. In Turkey, only two species, *M. lyra* and *M. subalpina*, of this genus have been known until now (Kazancı and Ertunç 2008). The pupal features of our specimens are similar to those of *M. subalpina*, reported from four localities in Anatolia, one in the northwestern and three in the eastern and southeastern parts of the country, by Crosskey and Zwick (2007). *Metacnephia subalpina* was described by Rubtsov (1956) as a variety of *M. nigra*, but Terteryan (1968) elevated it to species. According to Crosskey and Zwick (2007), the relationship of *M. subalpina* and *M. nigra* remains unsettled because of the problem of the true identity of *M. nigra*; the authors could not resolve the ambiguities. They noted that Yankovsky (1995) (pp. 4, 50) treated *subalpina* and *nigra* as synonyms before reverting [Yankovsky (2003)] to treating them as separate species. Crosskey and Zwick (2007) used the name *subalpina* for their material because it conformed in the shape of the ventral plate and pupal gill with Djafarov’s illustrations of *M. subalpina*. Another species, *Metacnephia uzunovi*, described by Kovachev (1985) from Bulgaria, confuses the taxonomic situation by having similar structural characters to *M. nigra*. We have doubts about its validity. We recognize *M. nigra* as a species complex, following the recommendation of P.H. Adler (pers. comm.) who noted the similarity with Rubtsov’s (1956, fig. 113) variety, *saxicola*, of *M. nigra*. Adler suggested that we use the name *M. nigra* for our material, with reference to Rubtsov’s (1956) variety *saxicola*, as a conservative approach that will provide future workers with an idea of the morphology of our material until the correct name of the species can be determined. *M. nigra* is known from Azerbaijan (Nakhichevan), Romania, Russia (Caucasus) and Turkmenistan (Adler and Crosskey 2015).

### Simulium (Boophthora) erythrocephalum

(De Geer, 1776)

#### Materials

- **Type status:** Other material. **Occurrence:** sex: 1 male pharate adult; lifeStage: 4 pupae, 3 larvae / 3 pupae, 1 larvae; **Taxon:** scientificName: Simulium (Boophthora) erythrocephalum (De Geer, 1776); kingdom: Animalia; phylum: Arthropoda; class: Insecta; order: Diptera; family: Simuliidae; genus: Simulium; subgenus: Boophthora; scientificNameAuthorship: (De Geer, 1776); **Location:** continent: Europe; country: Turkey; stateProvince: Tekirdağ; county: Saray; locationRemarks: 23; verbatimLatitude: 41°28'49.33"N; verbatimLongitude: 27°57'11.84"E; **Identification:** identificationID: esoguent-th-ıd-16; **Event:** eventDate: 24.05.2002 / 09.07.2003
- **Type status:** Other material. **Occurrence:** lifeStage: 4 pupae, 5 larvae; **Taxon:** scientificName: Simulium (Boophthora) erythrocephalum (De Geer, 1776); kingdom: Animalia; phylum: Arthropoda; class: Insecta; order: Diptera; family: Simuliidae; genus: Simulium; subgenus: Boophthora; scientificNameAuthorship: (De Geer, 1776); **Location:** continent: Europe; country: Turkey; stateProvince: Edirne; county: İnece; locationRemarks: 55; verbatimLatitude: 41°40'13.00"N; verbatimLongitude: 27°4'7.90"E; **Identification:** identificationID: esoguent-th-ıd-17; **Event:** eventDate: 05/29/2002
- **Type status:** Other material. **Occurrence:** lifeStage: 9 pupae, 6 larvae; **Taxon:** scientificName: Simulium (Boophthora) erythrocephalum (De Geer, 1776); kingdom: Animalia; phylum: Arthropoda; class: Insecta; order: Diptera; family: Simuliidae; genus: Simulium; subgenus: Boophthora; scientificNameAuthorship: (De Geer, 1776); **Location:** continent: Europe; country: Turkey; stateProvince: Edirne; county: İnece; locationRemarks: 56; verbatimLatitude: 41°40'42.77"N; verbatimLongitude: 26°59'24.21"E; **Identification:** identificationID: esoguent-th-ıd-18; **Event:** eventDate: 05/29/2002
- **Type status:** Other material. **Occurrence:** lifeStage: 8 pupae, 7 larvae; **Taxon:** scientificName: Simulium (Boophthora) erythrocephalum (De Geer, 1776); kingdom: Animalia; phylum: Arthropoda; class: Insecta; order: Diptera; family: Simuliidae; genus: Simulium; subgenus: Boophthora; scientificNameAuthorship: (De Geer, 1776); **Location:** continent: Europe; country: Turkey; stateProvince: Edirne; county: Merkez; locationRemarks: 57; verbatimLatitude: 41°44'12.71"N; verbatimLongitude: 26°39'26.00"E; **Identification:** identificationID: esoguent-th-ıd-19; **Event:** eventDate: 05/29/2002
- **Type status:** Other material. **Occurrence:** lifeStage: 6 pupae, 3 larvae; **Taxon:** scientificName: Simulium (Boophthora) erythrocephalum (De Geer, 1776); kingdom: Animalia; phylum: Arthropoda; class: Insecta; order: Diptera; family: Simuliidae; genus: Simulium; subgenus: Boophthora; scientificNameAuthorship: (De Geer, 1776); **Location:** continent: Europe; country: Turkey; stateProvince: Edirne; county: Merkez; locationRemarks: 61; verbatimLatitude: 41°43'25.74"N; verbatimLongitude: 26°33'38.19"E; **Identification:** identificationID: esoguent-th-ıd-20; **Event:** eventDate: 05/30/2002
- **Type status:** Other material. **Occurrence:** lifeStage: 4 pupae, 8 larvae; **Taxon:** scientificName: Simulium (Boophthora) erythrocephalum (De Geer, 1776); kingdom: Animalia; phylum: Arthropoda; class: Insecta; order: Diptera; family: Simuliidae; genus: Simulium; subgenus: Boophthora; scientificNameAuthorship: (De Geer, 1776); **Location:** continent: Europe; country: Turkey; stateProvince: Edirne; county: Hatipköy; locationRemarks: 63; verbatimLatitude: 41°48'14.99"N; verbatimLongitude: 26°33'13.31"E; **Identification:** identificationID: esoguent-th-ıd-21; **Event:** eventDate: 05/30/2002
- **Type status:** Other material. **Occurrence:** lifeStage: 8 pupae, 5 larvae; **Taxon:** scientificName: Simulium (Boophthora) erythrocephalum (De Geer, 1776); kingdom: Animalia; phylum: Arthropoda; class: Insecta; order: Diptera; family: Simuliidae; genus: Simulium; subgenus: Boophthora; scientificNameAuthorship: (De Geer, 1776); **Location:** continent: Europe; country: Turkey; stateProvince: Edirne; county: Havsa; locationRemarks: 67; verbatimLatitude: 41°22'5.91"N; verbatimLongitude: 26°40'1.43"E; **Identification:** identificationID: esoguent-th-ıd-22; **Event:** eventDate: 06/01/2002
- **Type status:** Other material. **Occurrence:** sex: 3 males, 3 females; lifeStage: 5 pupae, 7 larvae; **Taxon:** scientificName: Simulium (Boophthora) erythrocephalum (De Geer, 1776); kingdom: Animalia; phylum: Arthropoda; class: Insecta; order: Diptera; family: Simuliidae; genus: Simulium; subgenus: Boophthora; scientificNameAuthorship: (De Geer, 1776); **Location:** continent: Europe; country: Turkey; stateProvince: Edirne; county: Uzunköprü; locationRemarks: 68; verbatimLatitude: 41°10'9.16"N; verbatimLongitude: 26°40'17.11"E; **Identification:** identificationID: esoguent-th-ıd-23; **Event:** eventDate: 06/01/2002
- **Type status:** Other material. **Occurrence:** sex: 1 male pharate adult; lifeStage: 2 pupae, 9 larvae, 1 pupae; **Taxon:** scientificName: Simulium (Boophthora) erythrocephalum (De Geer, 1776); kingdom: Animalia; phylum: Arthropoda; class: Insecta; order: Diptera; family: Simuliidae; genus: Simulium; subgenus: Boophthora; scientificNameAuthorship: (De Geer, 1776); **Location:** continent: Europe; country: Turkey; stateProvince: Edirne; county: Uzunköprü; locationRemarks: 69; verbatimLatitude: 41°6'21.69"N; verbatimLongitude: 26°38'11.89"E; **Identification:** identificationID: esoguent-th-ıd-24; **Event:** eventDate: 01.06.2002 / 06.07.2003
- **Type status:** Other material. **Occurrence:** lifeStage: 6 pupae, 6 larvae; **Taxon:** scientificName: Simulium (Boophthora) erythrocephalum (De Geer, 1776); kingdom: Animalia; phylum: Arthropoda; class: Insecta; order: Diptera; family: Simuliidae; genus: Simulium; subgenus: Boophthora; scientificNameAuthorship: (De Geer, 1776); **Location:** continent: Europe; country: Turkey; stateProvince: Edirne; county: Keşan; locationRemarks: 70; verbatimLatitude: 41°5'0.44"N; verbatimLongitude: 26°32'11.32"E; **Identification:** identificationID: esoguent-th-ıd-25; **Event:** eventDate: 06/01/2002
- **Type status:** Other material. **Occurrence:** lifeStage: 4 pupae, 7 larvae; **Taxon:** scientificName: Simulium (Boophthora) erythrocephalum (De Geer, 1776); kingdom: Animalia; phylum: Arthropoda; class: Insecta; order: Diptera; family: Simuliidae; genus: Simulium; subgenus: Boophthora; scientificNameAuthorship: (De Geer, 1776); **Location:** continent: Europe; country: Turkey; stateProvince: Edirne; county: Keşan; locationRemarks: 75; verbatimLatitude: 40°46'5.97"N; verbatimLongitude: 26°30'42.72"E; **Identification:** identificationID: esoguent-th-ıd-26; **Event:** eventDate: 06/02/2002
- **Type status:** Other material. **Occurrence:** sex: 1 male pharate adult; lifeStage: 8 pupae, 7 larvae; **Taxon:** scientificName: Simulium (Boophthora) erythrocephalum (De Geer, 1776); kingdom: Animalia; phylum: Arthropoda; class: Insecta; order: Diptera; family: Simuliidae; genus: Simulium; subgenus: Boophthora; scientificNameAuthorship: (De Geer, 1776); **Location:** continent: Europe; country: Turkey; stateProvince: Edirne; county: Keşan; locationRemarks: 79; verbatimLatitude: 40°46'47.72"N; verbatimLongitude: 26°41'3.96"E; **Identification:** identificationID: esoguent-th-ıd-27; **Event:** eventDate: 06/03/2002
- **Type status:** Other material. **Occurrence:** lifeStage: 4 pupae / 2 pupae, 5 larvae; **Taxon:** scientificName: Simulium (Boophthora) erythrocephalum (De Geer, 1776); kingdom: Animalia; phylum: Arthropoda; class: Insecta; order: Diptera; family: Simuliidae; genus: Simulium; subgenus: Boophthora; scientificNameAuthorship: (De Geer, 1776); **Location:** continent: Europe; country: Turkey; stateProvince: Tekirdağ; county: Şarköy; locationRemarks: 83; verbatimLatitude: 40°46'21.52"N; verbatimLongitude: 27°3'32.09"E; **Identification:** identificationID: esoguent-th-ıd-28; **Event:** eventDate: 01.06.2002 / 05.07.2003
- **Type status:** Other material. **Occurrence:** lifeStage: 4 pupae, 8 larvae; **Taxon:** scientificName: Simulium (Boophthora) erythrocephalum (De Geer, 1776); kingdom: Animalia; phylum: Arthropoda; class: Insecta; order: Diptera; family: Simuliidae; genus: Simulium; subgenus: Boophthora; scientificNameAuthorship: (De Geer, 1776); **Location:** continent: Europe; country: Turkey; stateProvince: Tekirdağ; county: Malkara; locationRemarks: 93; verbatimLatitude: 40°56'45.42"N; verbatimLongitude: 26°53'8.36"E; **Identification:** identificationID: esoguent-th-ıd-29; **Event:** eventDate: 06/06/2002
- **Type status:** Other material. **Occurrence:** lifeStage: 5 pupae, 5 larvae; **Taxon:** scientificName: Simulium (Boophthora) erythrocephalum (De Geer, 1776); kingdom: Animalia; phylum: Arthropoda; class: Insecta; order: Diptera; family: Simuliidae; genus: Simulium; subgenus: Boophthora; scientificNameAuthorship: (De Geer, 1776); **Location:** continent: Europe; country: Turkey; stateProvince: Tekirdağ; county: Malkara; locationRemarks: 97; verbatimLatitude: 41°0'48.93"N; verbatimLongitude: 26°56'39.41"E; **Identification:** identificationID: esoguent-th-ıd-30; **Event:** eventDate: 06/06/2002
- **Type status:** Other material. **Occurrence:** sex: 1 male; lifeStage: 6 pupae, 3 larvae; **Taxon:** scientificName: Simulium (Boophthora) erythrocephalum (De Geer, 1776); kingdom: Animalia; phylum: Arthropoda; class: Insecta; order: Diptera; family: Simuliidae; genus: Simulium; subgenus: Boophthora; scientificNameAuthorship: (De Geer, 1776); **Location:** continent: Europe; country: Turkey; stateProvince: Tekirdağ; county: Hayrabolu; locationRemarks: 98; verbatimLatitude: 41°7'22.32"N; verbatimLongitude: 27°7'49.78"E; **Identification:** identificationID: esoguent-th-ıd-31; **Event:** eventDate: 06/06/2002
- **Type status:** Other material. **Occurrence:** lifeStage: 7 pupae, 9 larvae; **Taxon:** scientificName: Simulium (Boophthora) erythrocephalum (De Geer, 1776); kingdom: Animalia; phylum: Arthropoda; class: Insecta; order: Diptera; family: Simuliidae; genus: Simulium; subgenus: Boophthora; scientificNameAuthorship: (De Geer, 1776); **Location:** continent: Europe; country: Turkey; stateProvince: Tekirdağ; county: Hayrabolu; locationRemarks: 100; verbatimLatitude: 41°11'28.14"N; verbatimLongitude: 27°12'29.08"E; **Identification:** identificationID: esoguent-th-ıd-32; **Event:** eventDate: 06/06/2002
- **Type status:** Other material. **Occurrence:** sex: 1 male pharate adult; lifeStage: 8 pupae, 5 larvae; **Taxon:** scientificName: Simulium (Boophthora) erythrocephalum (De Geer, 1776); kingdom: Animalia; phylum: Arthropoda; class: Insecta; order: Diptera; family: Simuliidae; genus: Simulium; subgenus: Boophthora; scientificNameAuthorship: (De Geer, 1776); **Location:** continent: Europe; country: Turkey; stateProvince: Kırklareli; county: Babaeski; locationRemarks: 103; verbatimLatitude: 41°27'8.31"N; verbatimLongitude: 27°2'44.09"E; **Identification:** identificationID: esoguent-th-ıd-33; **Event:** eventDate: 06/07/2002

#### Notes

*Simulium erythrocephalum* is the most abundant species of the subgenus *Boophthora* in the western Palearctic and is reported from the Far East of Asia (Adler and Crosskey 2015). Breeding sites of *S. erythrocephalum* are generally weedy, lowland streams and rivers, such as the Danube (Germany, Serbia), the Spree (Germany), the Warta (Poland), and the Morava (Czech Republich). In Central Europe, it occurs in various flowing-water systems such as lowland streams, outflows from ponds, irrigation constructions and large rivers (Werner and Kampen 2012). It can develop large populations and become a serious pest and nuisance to humans and farm animals (Ignjatović-Ćupına et al. 2006) In Anatolia, it has been recorded by several authors. Sirin and Sahin (2005) found it at two sites in the Sakarya river basin for the first time in Turkey. Kazancı and Ertunç (2008) stated that *S. erythrocephalum* lives in the Büyük Menderes river basin in southwestern Anatolia. It is recorded by Gazyağcı and Aydenizöz (2011) from the Kızılırmak River in Kırıkkale Province. The latest record for *S. erythrocephalum* from Turkey was given by Şirin et al. (2014) from Sakarya and Kocaeli Provinces. According to the keys of Knoz (1965) and Bass (1998), the species can be identified by a pair of inconspicuous dorsolateral papillae on each segment of the larva, the pattern of the larval head capsule, the branching pattern of the six pupal gill filaments, and the shape of the pupal cocoon. The male genitalia of our specimens conform to the description by Rubtsov (1956)​.

### Simulium (Eusimulium) petricolum

(Rivosecchi, 1963)

#### Materials

- **Type status:** Other material. **Occurrence:** sex: 1 male pharate adult; lifeStage: 3 pupae; **Taxon:** scientificName: Simulium (Eusimulium) petricolum (Rivosecchi, 1963); kingdom: Animalia; phylum: Arthropoda; class: Insecta; order: Diptera; family: Simuliidae; genus: Simulium; subgenus: Eusimulium; scientificNameAuthorship: (Rivosecchi, 1963); **Location:** continent: Europe; country: Turkey; stateProvince: İstanbul; county: Alibeyköy; locationRemarks: 4; verbatimLatitude: 41°9'3.88"N; verbatimLongitude: 28°53'29.90"E; **Identification:** identificationID: esoguent-th-ıd-34; **Event:** eventDate: 05/20/2002
- **Type status:** Other material. **Occurrence:** sex: 1 male, 1 male pharate adult, 1 female pharate adult,; lifeStage: 5 pupae, 8 larvae; **Taxon:** scientificName: Simulium (Eusimulium) petricolum (Rivosecchi, 1963); kingdom: Animalia; phylum: Arthropoda; class: Insecta; order: Diptera; family: Simuliidae; genus: Simulium; subgenus: Eusimulium; scientificNameAuthorship: (Rivosecchi, 1963); **Location:** continent: Europe; country: Turkey; stateProvince: İstanbul; county: Alibeyköy; locationRemarks: 5; verbatimLatitude: 41°9'29.46"N; verbatimLongitude: 28°50'20.95"E; **Identification:** identificationID: esoguent-th-ıd-35; **Event:** eventDate: 05/21/2002
- **Type status:** Other material. **Occurrence:** sex: 2 males pharate adult, 1 female pharate adult; lifeStage: 7 pupae, 13 larvae; **Taxon:** scientificName: Simulium (Eusimulium) petricolum (Rivosecchi, 1963); kingdom: Animalia; phylum: Arthropoda; class: Insecta; order: Diptera; family: Simuliidae; genus: Simulium; subgenus: Eusimulium; scientificNameAuthorship: (Rivosecchi, 1963); **Location:** continent: Europe; country: Turkey; stateProvince: İstanbul; county: Çatalca; locationRemarks: 6; verbatimLatitude: 41°11'40.48"N; verbatimLongitude: 28°45'45.58"E; **Identification:** identificationID: esoguent-th-ıd-36; **Event:** eventDate: 05/21/2002
- **Type status:** Other material. **Occurrence:** sex: 1 male pharate adult, 2 females pharate adult; lifeStage: 6 pupae, 11 larvae; **Taxon:** scientificName: Simulium (Eusimulium) petricolum (Rivosecchi, 1963); kingdom: Animalia; phylum: Arthropoda; class: Insecta; order: Diptera; family: Simuliidae; genus: Simulium; subgenus: Eusimulium; scientificNameAuthorship: (Rivosecchi, 1963); **Location:** continent: Europe; country: Turkey; stateProvince: İstanbul; county: Çatalca; locationRemarks: 7; verbatimLatitude: 41°12'22.30"N; verbatimLongitude: 28°38'8.26"E; **Identification:** identificationID: esoguent-th-ıd-37; **Event:** eventDate: 05/21/2002
- **Type status:** Other material. **Occurrence:** sex: 1 male pharate adult, 1 female pharate adult; lifeStage: 12 pupae, 3 larvae; **Taxon:** scientificName: Simulium (Eusimulium) petricolum (Rivosecchi, 1963); kingdom: Animalia; phylum: Arthropoda; class: Insecta; order: Diptera; family: Simuliidae; genus: Simulium; subgenus: Eusimulium; scientificNameAuthorship: (Rivosecchi, 1963); **Location:** continent: Europe; country: Turkey; stateProvince: İstanbul; county: Çatalca; locationRemarks: 8; verbatimLatitude: 41°11'46.99"N; verbatimLongitude: 28°36'54.42"E; **Identification:** identificationID: esoguent-th-ıd-38; **Event:** eventDate: 05/21/2002
- **Type status:** Other material. **Occurrence:** sex: 3 males pharate adult; lifeStage: 5 pupae, 8 larvae / 1 pupae; **Taxon:** scientificName: Simulium (Eusimulium) petricolum (Rivosecchi, 1963); kingdom: Animalia; phylum: Arthropoda; class: Insecta; order: Diptera; family: Simuliidae; genus: Simulium; subgenus: Eusimulium; scientificNameAuthorship: (Rivosecchi, 1963); **Location:** continent: Europe; country: Turkey; stateProvince: Kırklareli; county: Vize; locationRemarks: 29; verbatimLatitude: 41°37'42.79"N; verbatimLongitude: 27°38'58.94"E; **Identification:** identificationID: esoguent-th-ıd-39; **Event:** eventDate: 25.05.2002 / 08.07.2003
- **Type status:** Other material. **Occurrence:** sex: 1 male pharate adult, 1 female pharate adult; lifeStage: 6 pupae, 3 larvae; **Taxon:** scientificName: Simulium (Eusimulium) petricolum (Rivosecchi, 1963); kingdom: Animalia; phylum: Arthropoda; class: Insecta; order: Diptera; family: Simuliidae; genus: Simulium; subgenus: Eusimulium; scientificNameAuthorship: (Rivosecchi, 1963); **Location:** continent: Europe; country: Turkey; stateProvince: Kırklareli; county: Üsküp; locationRemarks: 39; verbatimLatitude: 41°43'1.19"N; verbatimLongitude: 27°22'20.34"E; **Identification:** identificationID: esoguent-th-ıd-40; **Event:** eventDate: 05/27/2002
- **Type status:** Other material. **Occurrence:** sex: 2 males pharate adult; lifeStage: 10 pupae, 14 larvae / 5 pupae, 8 larvae; **Taxon:** scientificName: Simulium (Eusimulium) petricolum (Rivosecchi, 1963); kingdom: Animalia; phylum: Arthropoda; class: Insecta; order: Diptera; family: Simuliidae; genus: Simulium; subgenus: Eusimulium; scientificNameAuthorship: (Rivosecchi, 1963); **Location:** continent: Europe; country: Turkey; stateProvince: Kırklareli; county: Merkez; locationRemarks: 46; verbatimLatitude: 41°49'48.43"N; verbatimLongitude: 27°10'42.01"E; **Identification:** identificationID: esoguent-th-ıd-41; **Event:** eventDate: 28.05.2002 / 23.04.2005
- **Type status:** Other material. **Occurrence:** sex: 1 female pharate adult; lifeStage: 3 pupae; **Taxon:** scientificName: Simulium (Eusimulium) petricolum (Rivosecchi, 1963); kingdom: Animalia; phylum: Arthropoda; class: Insecta; order: Diptera; family: Simuliidae; genus: Simulium; subgenus: Eusimulium; scientificNameAuthorship: (Rivosecchi, 1963); **Location:** continent: Europe; country: Turkey; stateProvince: Kırklareli; county: Kofçaz; locationRemarks: 47; verbatimLatitude: 41°52'37.87"N; verbatimLongitude: 27°10'41.95"E; **Identification:** identificationID: esoguent-th-ıd-42; **Event:** eventDate: 05/28/2002
- **Type status:** Other material. **Occurrence:** sex: 1 female pharate adult; lifeStage: 8 pupae; **Taxon:** scientificName: Simulium (Eusimulium) petricolum (Rivosecchi, 1963); kingdom: Animalia; phylum: Arthropoda; class: Insecta; order: Diptera; family: Simuliidae; genus: Simulium; subgenus: Eusimulium; scientificNameAuthorship: (Rivosecchi, 1963); **Location:** continent: Europe; country: Turkey; stateProvince: Kırklareli; county: Kofçaz; locationRemarks: 48; verbatimLatitude: 41°54'8.88"N; verbatimLongitude: 27°10'30.90"E; **Identification:** identificationID: esoguent-th-ıd-43; **Event:** eventDate: 05/28/2002
- **Type status:** Other material. **Occurrence:** sex: 1 female, 1 male pharate adult; lifeStage: 6 pupae, 9 larvae / 2 pupae,; **Taxon:** scientificName: Simulium (Eusimulium) petricolum (Rivosecchi, 1963); kingdom: Animalia; phylum: Arthropoda; class: Insecta; order: Diptera; family: Simuliidae; genus: Simulium; subgenus: Eusimulium; scientificNameAuthorship: (Rivosecchi, 1963); **Location:** continent: Europe; country: Turkey; stateProvince: Kırklareli; county: Kofçaz; locationRemarks: 49; verbatimLatitude: 41°55'33.95"N; verbatimLongitude: 27°9'48.15"E; **Identification:** identificationID: esoguent-th-ıd-44; **Event:** eventDate: 28.05.2002 / 23.04.2005
- **Type status:** Other material. **Occurrence:** sex: 2 males pharate adult; lifeStage: 5 pupae; **Taxon:** scientificName: Simulium (Eusimulium) petricolum (Rivosecchi, 1963); kingdom: Animalia; phylum: Arthropoda; class: Insecta; order: Diptera; family: Simuliidae; genus: Simulium; subgenus: Eusimulium; scientificNameAuthorship: (Rivosecchi, 1963); **Location:** continent: Europe; country: Turkey; stateProvince: Edirne; county: Lalapaşa; locationRemarks: 59; verbatimLatitude: 41°51'12.74"N; verbatimLongitude: 26°43'20.06"E; **Identification:** identificationID: esoguent-th-ıd-45; **Event:** eventDate: 05/29/2002
- **Type status:** Other material. **Occurrence:** sex: 1 male, 1 male pharate adult; lifeStage: 7 pupae, 5 larvae; **Taxon:** scientificName: Simulium (Eusimulium) petricolum (Rivosecchi, 1963); kingdom: Animalia; phylum: Arthropoda; class: Insecta; order: Diptera; family: Simuliidae; genus: Simulium; subgenus: Eusimulium; scientificNameAuthorship: (Rivosecchi, 1963); **Location:** continent: Europe; country: Turkey; stateProvince: Edirne; county: Lalapaşa; locationRemarks: 60; verbatimLatitude: 41°50'24.70"N; verbatimLongitude: 26°36'30.45"E; **Identification:** identificationID: esoguent-th-ıd-46; **Event:** eventDate: 05/29/2002
- **Type status:** Other material. **Occurrence:** sex: 1 male pharate adult, 1 female pharate adult; lifeStage: 5 pupae, 4 larvae; **Taxon:** scientificName: Simulium (Eusimulium) petricolum (Rivosecchi, 1963); kingdom: Animalia; phylum: Arthropoda; class: Insecta; order: Diptera; family: Simuliidae; genus: Simulium; subgenus: Eusimulium; scientificNameAuthorship: (Rivosecchi, 1963); **Location:** continent: Europe; country: Turkey; stateProvince: Edirne; county: Havsa; locationRemarks: 66; verbatimLatitude: 41°21'20.92"N; verbatimLongitude: 26°44'25.80"E; **Identification:** identificationID: esoguent-th-ıd-47; **Event:** eventDate: 05/31/2002
- **Type status:** Other material. **Occurrence:** sex: 1 male pharate adult; lifeStage: 6 pupae, 11 larvae; **Taxon:** scientificName: Simulium (Eusimulium) petricolum (Rivosecchi, 1963); kingdom: Animalia; phylum: Arthropoda; class: Insecta; order: Diptera; family: Simuliidae; genus: Simulium; subgenus: Eusimulium; scientificNameAuthorship: (Rivosecchi, 1963); **Location:** continent: Europe; country: Turkey; stateProvince: Edirne; county: Havsa; locationRemarks: 67; verbatimLatitude: 41°22'5.91"N; verbatimLongitude: 26°40'1.43"E; **Identification:** identificationID: esoguent-th-ıd-48; **Event:** eventDate: 06/01/2002
- **Type status:** Other material. **Occurrence:** sex: 1 males pharate adult; lifeStage: 3 pupae, 7 larvae / 3 pupae, 2 larvae; **Taxon:** scientificName: Simulium (Eusimulium) petricolum (Rivosecchi, 1963); kingdom: Animalia; phylum: Arthropoda; class: Insecta; order: Diptera; family: Simuliidae; genus: Simulium; subgenus: Eusimulium; scientificNameAuthorship: (Rivosecchi, 1963); **Location:** continent: Europe; country: Turkey; stateProvince: Edirne; county: Uzunköprü; locationRemarks: 69; verbatimLatitude: 41°6'21.69"N; verbatimLongitude: 26°38'11.89"E; **Identification:** identificationID: esoguent-th-ıd-49; **Event:** eventDate: 01.06.2002 / 23.04.2005
- **Type status:** Other material. **Occurrence:** sex: 1 male pharate adult, 1 female pharate adult; lifeStage: 4 pupae, 7 larvae; **Taxon:** scientificName: Simulium (Eusimulium) petricolum (Rivosecchi, 1963); kingdom: Animalia; phylum: Arthropoda; class: Insecta; order: Diptera; family: Simuliidae; genus: Simulium; subgenus: Eusimulium; scientificNameAuthorship: (Rivosecchi, 1963); **Location:** continent: Europe; country: Turkey; stateProvince: Edirne; county: Keşan; locationRemarks: 74; verbatimLatitude: 40°56'4.00"N; verbatimLongitude: 26°34'9.15"E; **Identification:** identificationID: esoguent-th-ıd-50; **Event:** eventDate: 06/01/2002
- **Type status:** Other material. **Occurrence:** sex: 1 male pharate adult; lifeStage: 7 pupae; **Taxon:** scientificName: Simulium (Eusimulium) petricolum (Rivosecchi, 1963); kingdom: Animalia; phylum: Arthropoda; class: Insecta; order: Diptera; family: Simuliidae; genus: Simulium; subgenus: Eusimulium; scientificNameAuthorship: (Rivosecchi, 1963); **Location:** continent: Europe; country: Turkey; stateProvince: Edirne; county: Keşan; locationRemarks: 76; verbatimLatitude: 40°43'58.64"N; verbatimLongitude: 26°26'11.64"E; **Identification:** identificationID: esoguent-th-ıd-51; **Event:** eventDate: 06/02/2002
- **Type status:** Other material. **Occurrence:** sex: 1 male pharate adult; lifeStage: 5 pupae, 6 larvae; **Taxon:** scientificName: Simulium (Eusimulium) petricolum (Rivosecchi, 1963); kingdom: Animalia; phylum: Arthropoda; class: Insecta; order: Diptera; family: Simuliidae; genus: Simulium; subgenus: Eusimulium; scientificNameAuthorship: (Rivosecchi, 1963); **Location:** continent: Europe; country: Turkey; stateProvince: Edirne; county: Enez; locationRemarks: 77; verbatimLatitude: 40°39'18.66"N; verbatimLongitude: 26°13'21.15"E; **Identification:** identificationID: esoguent-th-ıd-52; **Event:** eventDate: 06/02/2002
- **Type status:** Other material. **Occurrence:** sex: 1 male pharate adult; lifeStage: 5 pupae, 10 larvae; **Taxon:** scientificName: Simulium (Eusimulium) petricolum (Rivosecchi, 1963); kingdom: Animalia; phylum: Arthropoda; class: Insecta; order: Diptera; family: Simuliidae; genus: Simulium; subgenus: Eusimulium; scientificNameAuthorship: (Rivosecchi, 1963); **Location:** continent: Europe; country: Turkey; stateProvince: Edirne; county: Enez; locationRemarks: 78; verbatimLatitude: 40°40'25.25"N; verbatimLongitude: 26°11'17.04"E; **Identification:** identificationID: esoguent-th-ıd-53; **Event:** eventDate: 06/02/2002
- **Type status:** Other material. **Occurrence:** sex: 1 male pharate adult; lifeStage: 3 pupae, 8 larvae; **Taxon:** scientificName: Simulium (Eusimulium) petricolum (Rivosecchi, 1963); kingdom: Animalia; phylum: Arthropoda; class: Insecta; order: Diptera; family: Simuliidae; genus: Simulium; subgenus: Eusimulium; scientificNameAuthorship: (Rivosecchi, 1963); **Location:** continent: Europe; country: Turkey; stateProvince: Edirne; county: Keşan; locationRemarks: 79; verbatimLatitude: 40°46'47.72"N; verbatimLongitude: 26°41'3.96"E; **Identification:** identificationID: esoguent-th-ıd-54; **Event:** eventDate: 06/03/2002
- **Type status:** Other material. **Occurrence:** sex: 1 male,1 male pharate adult; lifeStage: 4 pupae, 6 larvae; **Taxon:** scientificName: Simulium (Eusimulium) petricolum (Rivosecchi, 1963); kingdom: Animalia; phylum: Arthropoda; class: Insecta; order: Diptera; family: Simuliidae; genus: Simulium; subgenus: Eusimulium; scientificNameAuthorship: (Rivosecchi, 1963); **Location:** continent: Europe; country: Turkey; stateProvince: Edirne; county: Keşan; locationRemarks: 80; verbatimLatitude: 40°43'36.00"N; verbatimLongitude: 26°42'46.33"E; **Identification:** identificationID: esoguent-th-ıd-55; **Event:** eventDate: 06/03/2002
- **Type status:** Other material. **Occurrence:** sex: 1 male, 1 female, 1 male pharate adult; lifeStage: 3 pupae, 8 larvae; **Taxon:** scientificName: Simulium (Eusimulium) petricolum (Rivosecchi, 1963); kingdom: Animalia; phylum: Arthropoda; class: Insecta; order: Diptera; family: Simuliidae; genus: Simulium; subgenus: Eusimulium; scientificNameAuthorship: (Rivosecchi, 1963); **Location:** continent: Europe; country: Turkey; stateProvince: Çanakkale; county: Gelibolu; locationRemarks: 81; verbatimLatitude: 40°39'38.12"N; verbatimLongitude: 26°49'14.41"E; **Identification:** identificationID: esoguent-th-ıd-56; **Event:** eventDate: 06/03/2002
- **Type status:** Other material. **Occurrence:** sex: 1 male, 1 female, 3 males pharate adult; lifeStage: 5 pupae, 1 larvae / 7 pupae, 7 larvae; **Taxon:** scientificName: Simulium (Eusimulium) petricolum (Rivosecchi, 1963); kingdom: Animalia; phylum: Arthropoda; class: Insecta; order: Diptera; family: Simuliidae; genus: Simulium; subgenus: Eusimulium; scientificNameAuthorship: (Rivosecchi, 1963); **Location:** continent: Europe; country: Turkey; stateProvince: Tekirdağ; county: Şarköy; locationRemarks: 82; verbatimLatitude: 40°41'28.95"N; verbatimLongitude: 27°5'45.01"E; **Identification:** identificationID: esoguent-th-ıd-57; **Event:** eventDate: 04.06.2002 / 05.07.2003
- **Type status:** Other material. **Occurrence:** sex: 2 males pharate adult, 2 females pharate adult; lifeStage: 4 pupae, 7 larvae / 2 pupae; **Taxon:** scientificName: Simulium (Eusimulium) petricolum (Rivosecchi, 1963); kingdom: Animalia; phylum: Arthropoda; class: Insecta; order: Diptera; family: Simuliidae; genus: Simulium; subgenus: Eusimulium; scientificNameAuthorship: (Rivosecchi, 1963); **Location:** continent: Europe; country: Turkey; stateProvince: Tekirdağ; county: Şarköy; locationRemarks: 83; verbatimLatitude: 40°46'21.52"N; verbatimLongitude: 27°3'32.09"E; **Identification:** identificationID: esoguent-th-ıd-58; **Event:** eventDate: 04.06.2002 / 05.07.2003
- **Type status:** Other material. **Occurrence:** sex: 1 male, 1 male pharate adult; lifeStage: 3 pupae, 6 larvae; **Taxon:** scientificName: Simulium (Eusimulium) petricolum (Rivosecchi, 1963); kingdom: Animalia; phylum: Arthropoda; class: Insecta; order: Diptera; family: Simuliidae; genus: Simulium; subgenus: Eusimulium; scientificNameAuthorship: (Rivosecchi, 1963); **Location:** continent: Europe; country: Turkey; stateProvince: Tekirdağ; county: Malkara; locationRemarks: 84; verbatimLatitude: 40°48'44.49"N; verbatimLongitude: 26°59'30.47"E; **Identification:** identificationID: esoguent-th-ıd-59; **Event:** eventDate: 06/06/2002
- **Type status:** Other material. **Occurrence:** sex: 2 males pharate adult; lifeStage: 7 pupae, 4 larvae; **Taxon:** scientificName: Simulium (Eusimulium) petricolum (Rivosecchi, 1963); kingdom: Animalia; phylum: Arthropoda; class: Insecta; order: Diptera; family: Simuliidae; genus: Simulium; subgenus: Eusimulium; scientificNameAuthorship: (Rivosecchi, 1963); **Location:** continent: Europe; country: Turkey; stateProvince: Tekirdağ; county: Malkara; locationRemarks: 85; verbatimLatitude: 40°48'30.46"N; verbatimLongitude: 26°56'23.51"E; **Identification:** identificationID: esoguent-th-ıd-60; **Event:** eventDate: 06/04/2002
- **Type status:** Other material. **Occurrence:** sex: 1 male, 1 male pharate adult; lifeStage: 9 pupae, 12 larvae; **Taxon:** scientificName: Simulium (Eusimulium) petricolum (Rivosecchi, 1963); kingdom: Animalia; phylum: Arthropoda; class: Insecta; order: Diptera; family: Simuliidae; genus: Simulium; subgenus: Eusimulium; scientificNameAuthorship: (Rivosecchi, 1963); **Location:** continent: Europe; country: Turkey; stateProvince: Tekirdağ; county: Malkara; locationRemarks: 87; verbatimLatitude: 40°47'19.76"; verbatimLongitude: 26°48'15.30"E; **Identification:** identificationID: esoguent-th-ıd-61; **Event:** eventDate: 06/04/2002
- **Type status:** Other material. **Occurrence:** sex: 1 male pharate adult, 2 females pharate adult; lifeStage: 5 pupae, 6 larvae; **Taxon:** scientificName: Simulium (Eusimulium) petricolum (Rivosecchi, 1963); kingdom: Animalia; phylum: Arthropoda; class: Insecta; order: Diptera; family: Simuliidae; genus: Simulium; subgenus: Eusimulium; scientificNameAuthorship: (Rivosecchi, 1963); **Location:** continent: Europe; country: Turkey; stateProvince: Çanakkale; county: Gelibolu; locationRemarks: 88; verbatimLatitude: 40°41'38.58"N; verbatimLongitude: 26°55'56.44"E; **Identification:** identificationID: esoguent-th-ıd-62; **Event:** eventDate: 06/04/2002
- **Type status:** Other material. **Occurrence:** sex: 2 males pharate adult; lifeStage: 8 pupae, 11 larvae; **Taxon:** scientificName: Simulium (Eusimulium) petricolum (Rivosecchi, 1963); kingdom: Animalia; phylum: Arthropoda; class: Insecta; order: Diptera; family: Simuliidae; genus: Simulium; subgenus: Eusimulium; scientificNameAuthorship: (Rivosecchi, 1963); **Location:** continent: Europe; country: Turkey; stateProvince: Çanakkale; county: Gelibolu; locationRemarks: 89; verbatimLatitude: 40°42'4.87"N; verbatimLongitude: 26°54'53.59"E; **Identification:** identificationID: esoguent-th-ıd-63; **Event:** eventDate: 06/04/2002
- **Type status:** Other material. **Occurrence:** sex: 1 male pharate adult, 1 female pharate adult; lifeStage: 5 pupae; **Taxon:** scientificName: Simulium (Eusimulium) petricolum (Rivosecchi, 1963); kingdom: Animalia; phylum: Arthropoda; class: Insecta; order: Diptera; family: Simuliidae; genus: Simulium; subgenus: Eusimulium; scientificNameAuthorship: (Rivosecchi, 1963); **Location:** continent: Europe; country: Turkey; stateProvince: Tekirdağ; county: Malkara; locationRemarks: 93; verbatimLatitude: 40°56'45.42"N; verbatimLongitude: 26°53'8.36"E; **Identification:** identificationID: esoguent-th-ıd-64; **Event:** eventDate: 06/06/2002
- **Type status:** Other material. **Occurrence:** sex: 1 male, 1 male pharate adult; lifeStage: 3 pupae; **Taxon:** scientificName: Simulium (Eusimulium) petricolum (Rivosecchi, 1963); kingdom: Animalia; phylum: Arthropoda; class: Insecta; order: Diptera; family: Simuliidae; genus: Simulium; subgenus: Eusimulium; scientificNameAuthorship: (Rivosecchi, 1963); **Location:** continent: Europe; country: Turkey; stateProvince: Tekirdağ; county: Malkara; locationRemarks: 96; verbatimLatitude: 40°58'35.77"N; verbatimLongitude: 26°54'37.73"E; **Identification:** identificationID: esoguent-th-ıd-65; **Event:** eventDate: 06/06/2002
- **Type status:** Other material. **Occurrence:** sex: 1 male pharate adult, 1 female pharate adult; lifeStage: 4 pupae, 5 larvae; **Taxon:** scientificName: Simulium (Eusimulium) petricolum (Rivosecchi, 1963); kingdom: Animalia; phylum: Arthropoda; class: Insecta; order: Diptera; family: Simuliidae; genus: Simulium; subgenus: Eusimulium; scientificNameAuthorship: (Rivosecchi, 1963); **Location:** continent: Europe; country: Turkey; stateProvince: Tekirdağ; county: Malkara; locationRemarks: 97; verbatimLatitude: 41°0'48.93"N; verbatimLongitude: 26°56'39.41"E; **Identification:** identificationID: esoguent-th-ıd-66; **Event:** eventDate: 06/06/2002
- **Type status:** Other material. **Occurrence:** sex: 1 male, 1 female, 2 males pharate adult; lifeStage: 4 pupae; **Taxon:** scientificName: Simulium (Eusimulium) petricolum (Rivosecchi, 1963); kingdom: Animalia; phylum: Arthropoda; class: Insecta; order: Diptera; family: Simuliidae; genus: Simulium; subgenus: Eusimulium; scientificNameAuthorship: (Rivosecchi, 1963); **Location:** continent: Europe; country: Turkey; stateProvince: Tekirdağ; county: Hayrabolu; locationRemarks: 99; verbatimLatitude: 41°10'16.80"N; verbatimLongitude: 27°3'37.78"E; **Identification:** identificationID: esoguent-th-ıd-67; **Event:** eventDate: 06/06/2002
- **Type status:** Other material. **Occurrence:** sex: 1 male pharate adult, 1 female pharate adult; lifeStage: 6 pupae, 7 larvae; **Taxon:** scientificName: Simulium (Eusimulium) petricolum (Rivosecchi, 1963); kingdom: Animalia; phylum: Arthropoda; class: Insecta; order: Diptera; family: Simuliidae; genus: Simulium; subgenus: Eusimulium; scientificNameAuthorship: (Rivosecchi, 1963); **Location:** continent: Europe; country: Turkey; stateProvince: Kırklareli; county: Babaeski; locationRemarks: 102; verbatimLatitude: 41°30'1.62"N; verbatimLongitude: 26°56'48.07"E; **Identification:** identificationID: esoguent-th-ıd-68; **Event:** eventDate: 06/07/2002
- **Type status:** Other material. **Occurrence:** sex: 1 male pharate adult; lifeStage: 3 pupae, 5 larvae; **Taxon:** scientificName: Simulium (Eusimulium) petricolum (Rivosecchi, 1963); kingdom: Animalia; phylum: Arthropoda; class: Insecta; order: Diptera; family: Simuliidae; genus: Simulium; subgenus: Eusimulium; scientificNameAuthorship: (Rivosecchi, 1963); **Location:** continent: Europe; country: Turkey; stateProvince: Tekirdağ; county: Kumbağ; locationRemarks: 112; verbatimLatitude: 40°51'7.69"N; verbatimLongitude: 27°20'3.10"E; **Identification:** identificationID: esoguent-th-ıd-69; **Event:** eventDate: 06/09/2002
- **Type status:** Other material. **Occurrence:** sex: 1 female pharate adult; lifeStage: 4 pupae, 8 larvae; **Taxon:** scientificName: Simulium (Eusimulium) petricolum (Rivosecchi, 1963); kingdom: Animalia; phylum: Arthropoda; class: Insecta; order: Diptera; family: Simuliidae; genus: Simulium; subgenus: Eusimulium; scientificNameAuthorship: (Rivosecchi, 1963); **Location:** continent: Europe; country: Turkey; stateProvince: Tekirdağ; county: Kumbağ; locationRemarks: 113; verbatimLatitude: 40°53'40.86"N; verbatimLongitude: 27°16'37.57"E; **Identification:** identificationID: esoguent-th-ıd-70; **Event:** eventDate: 06/09/2002
- **Type status:** Other material. **Occurrence:** sex: 1 male, 1 male pharate adult; lifeStage: 7 pupae, 5 larvae; **Taxon:** scientificName: Simulium (Eusimulium) petricolum (Rivosecchi, 1963); kingdom: Animalia; phylum: Arthropoda; class: Insecta; order: Diptera; family: Simuliidae; genus: Simulium; subgenus: Eusimulium; scientificNameAuthorship: (Rivosecchi, 1963); **Location:** continent: Europe; country: Turkey; stateProvince: Tekirdağ; county: Merkez; locationRemarks: 118; verbatimLatitude: 40°59'23.18"N; verbatimLongitude: 27°18'36.39"E; **Identification:** identificationID: esoguent-th-ıd-71; **Event:** eventDate: 06/10/2002
- **Type status:** Other material. **Occurrence:** sex: 1 male pharate adult, 1 female pharate adult; lifeStage: 6 pupae, 9 larvae; **Taxon:** scientificName: Simulium (Eusimulium) petricolum (Rivosecchi, 1963); kingdom: Animalia; phylum: Arthropoda; class: Insecta; order: Diptera; family: Simuliidae; genus: Simulium; subgenus: Eusimulium; scientificNameAuthorship: (Rivosecchi, 1963); **Location:** continent: Europe; country: Turkey; stateProvince: Tekirdağ; county: Merkez; locationRemarks: 119; verbatimLatitude: 41°2'17.26"N; verbatimLongitude: 27°19'6.35"E; **Identification:** identificationID: esoguent-th-ıd-72; **Event:** eventDate: 06/10/2002
- **Type status:** Other material. **Occurrence:** sex: 1 male, 2 males pharate adult; lifeStage: 8 pupae, 11 larvae; **Taxon:** scientificName: Simulium (Eusimulium) petricolum (Rivosecchi, 1963); kingdom: Animalia; phylum: Arthropoda; class: Insecta; order: Diptera; family: Simuliidae; genus: Simulium; subgenus: Eusimulium; scientificNameAuthorship: (Rivosecchi, 1963); **Location:** continent: Europe; country: Turkey; stateProvince: Tekirdağ; county: Muratlı; locationRemarks: 120; verbatimLatitude: 41°7'50.39"N; verbatimLongitude: 27°26'7.28"E; **Identification:** identificationID: esoguent-th-ıd-73; **Event:** eventDate: 06/10/2002
- **Type status:** Other material. **Occurrence:** sex: 1 male pharate adult; lifeStage: 7 pupae; **Taxon:** scientificName: Simulium (Eusimulium) petricolum (Rivosecchi, 1963); kingdom: Animalia; phylum: Arthropoda; class: Insecta; order: Diptera; family: Simuliidae; genus: Simulium; subgenus: Eusimulium; scientificNameAuthorship: (Rivosecchi, 1963); **Location:** continent: Europe; country: Turkey; stateProvince: Tekirdağ; county: Marmara Ereğlisi; locationRemarks: 121; verbatimLatitude: 41°4'27.74"N; verbatimLongitude: 27°54'40.09"E; **Identification:** identificationID: esoguent-th-ıd-74; **Event:** eventDate: 06/11/2002
- **Type status:** Other material. **Occurrence:** sex: 3 males pharate adult; lifeStage: 6 pupae, 8 larvae; **Taxon:** scientificName: Simulium (Eusimulium) petricolum (Rivosecchi, 1963); kingdom: Animalia; phylum: Arthropoda; class: Insecta; order: Diptera; family: Simuliidae; genus: Simulium; subgenus: Eusimulium; scientificNameAuthorship: (Rivosecchi, 1963); **Location:** continent: Europe; country: Turkey; stateProvince: İstanbul; county: Büyükçekmece; locationRemarks: 123; verbatimLatitude: 41°3'23.68"N; verbatimLongitude: 28°26'4.40"E; **Identification:** identificationID: esoguent-th-ıd-75; **Event:** eventDate: 06/11/2002

#### Notes

*Simulium petricolum* is one of three species in the *S. aureum* species group of the subgenus *Eusimulium* in Anatolia. The others are *S. angustipes* and *S. velutinum*. The first record of *S. petricolum* from Turkey was published by Kalafat and Şirin (2011). Gazyağcı and Aydenizöz (2011) recorded it from the Kızılırmak River in Kırıkkale Province. It was also reported from eastern Marmara Region by Şirin et al. (2014). *Simulium petricolum* occurs in southern Europe and especially in the Mediterranean countries (Crosskey and Malicky 2001). According to Crosskey and Malicky (2001), the species is not reliably distinguished from *S. velutinum* in the aquatic stages, adults being required for positive identification. Our specimens conform in genitalia to the descriptions by Crosskey and Malicky (2001) and Day et al. (2010)

### Simulium (Eusimulium) velutinum

(Santos Abreu, 1922)

#### Materials

- **Type status:** Other material. **Occurrence:** sex: 1 male pharate adult, 2 females pharate adult; lifeStage: 7 pupae, 5 larvae; **Taxon:** scientificName: Simulium (Eusimulium) velutinum (Santos Abreu, 1922); kingdom: Animalia; phylum: Arthropoda; class: Insecta; order: Diptera; family: Simuliidae; genus: Simulium; subgenus: Eusimulium; scientificNameAuthorship: (Santos Abreu, 1922); **Location:** continent: Europe; country: Turkey; stateProvince: İstanbul; county: Çatalca; locationRemarks: 10; verbatimLatitude: 41°13'57.35"N; verbatimLongitude: 28°30'15.57"E; **Identification:** identificationID: esoguent-th-ıd-76; **Event:** eventDate: 05/22/2002
- **Type status:** Other material. **Occurrence:** sex: 1 female pharate adult; lifeStage: 7 pupae, 4 larvae; **Taxon:** scientificName: Simulium (Eusimulium) velutinum (Santos Abreu, 1922); kingdom: Animalia; phylum: Arthropoda; class: Insecta; order: Diptera; family: Simuliidae; genus: Simulium; subgenus: Eusimulium; scientificNameAuthorship: (Santos Abreu, 1922); **Location:** continent: Europe; country: Turkey; stateProvince: İstanbul; county: Çatalca; locationRemarks: 11; verbatimLatitude: 41°14'51.86"N; verbatimLongitude: 28°25'50.19"E; **Identification:** identificationID: esoguent-th-ıd-77; **Event:** eventDate: 05/22/2002
- **Type status:** Other material. **Occurrence:** sex: 2 males, 2 females, 1 male pharate adult, 1 female pharate adult; lifeStage: 5 pupae, 8 larvae; **Taxon:** scientificName: Simulium (Eusimulium) velutinum (Santos Abreu, 1922); kingdom: Animalia; phylum: Arthropoda; class: Insecta; order: Diptera; family: Simuliidae; genus: Simulium; subgenus: Eusimulium; scientificNameAuthorship: (Santos Abreu, 1922); **Location:** continent: Europe; country: Turkey; stateProvince: Kırklareli; county: Pınarhisar; locationRemarks: 38; verbatimLatitude: 41°40'11.14"N; verbatimLongitude: 27°26'32.75"E; **Identification:** identificationID: esoguent-th-ıd-78; **Event:** eventDate: 05/27/2002
- **Type status:** Other material. **Occurrence:** sex: 1 male, 2 male pharate adult, 3 females pharate adult; lifeStage: 3 pupae, 12 larvae; **Taxon:** scientificName: Simulium (Eusimulium) velutinum (Santos Abreu, 1922); kingdom: Animalia; phylum: Arthropoda; class: Insecta; order: Diptera; family: Simuliidae; genus: Simulium; subgenus: Eusimulium; scientificNameAuthorship: (Santos Abreu, 1922); **Location:** continent: Europe; country: Turkey; stateProvince: Kırklareli; county: Üsküp; locationRemarks: 40; verbatimLatitude: 41°44'42.71"N; verbatimLongitude: 27°24'13.09"E; **Identification:** identificationID: esoguent-th-ıd-79; **Event:** eventDate: 05/27/2002
- **Type status:** Other material. **Occurrence:** sex: 1 male pharate adult, 2 females pharate adult; lifeStage: 6 pupae, 4 larvae / 6 pupae, 12 larvae,; **Taxon:** scientificName: Simulium (Eusimulium) velutinum (Santos Abreu, 1922); kingdom: Animalia; phylum: Arthropoda; class: Insecta; order: Diptera; family: Simuliidae; genus: Simulium; subgenus: Eusimulium; scientificNameAuthorship: (Santos Abreu, 1922); **Location:** continent: Europe; country: Turkey; stateProvince: Edirne; county: Lalapaşa; locationRemarks: 53; verbatimLatitude: 41°58'26.47"N; verbatimLongitude: 27°0'28.78"E; **Identification:** identificationID: esoguent-th-ıd-80; **Event:** eventDate: 28.05.2002 / 23.04.2005
- **Type status:** Other material. **Occurrence:** sex: 2 males pharate adult, 3 females pharate adult; lifeStage: 8 pupae, 6 larvae; **Taxon:** scientificName: Simulium (Eusimulium) velutinum (Santos Abreu, 1922); kingdom: Animalia; phylum: Arthropoda; class: Insecta; order: Diptera; family: Simuliidae; genus: Simulium; subgenus: Eusimulium; scientificNameAuthorship: (Santos Abreu, 1922); **Location:** continent: Europe; country: Turkey; stateProvince: Edirne; county: Lalapaşa; locationRemarks: 54; verbatimLatitude: 41°55'44.34"N; verbatimLongitude: 26°51'44.57"E; **Identification:** identificationID: esoguent-th-ıd-81; **Event:** eventDate: 05/28/2002
- **Type status:** Other material. **Occurrence:** sex: 1 male pharate adult, 2 females pharate adult; lifeStage: 7 pupae, 9 larvae; **Taxon:** scientificName: Simulium (Eusimulium) velutinum (Santos Abreu, 1922); kingdom: Animalia; phylum: Arthropoda; class: Insecta; order: Diptera; family: Simuliidae; genus: Simulium; subgenus: Eusimulium; scientificNameAuthorship: (Santos Abreu, 1922); **Location:** continent: Europe; country: Turkey; stateProvince: Edirne; county: Keşan; locationRemarks: 70; verbatimLatitude: 41°5'0.44"N; verbatimLongitude: 26°32'11.32"E; **Identification:** identificationID: esoguent-th-ıd-82; **Event:** eventDate: 06/01/2002
- **Type status:** Other material. **Occurrence:** sex: 2 males pharate adult; lifeStage: 5 pupae, 7 larvae; **Taxon:** scientificName: Simulium (Eusimulium) velutinum (Santos Abreu, 1922); kingdom: Animalia; phylum: Arthropoda; class: Insecta; order: Diptera; family: Simuliidae; genus: Simulium; subgenus: Eusimulium; scientificNameAuthorship: (Santos Abreu, 1922); **Location:** continent: Europe; country: Turkey; stateProvince: Çanakkale; county: Gelibolu; locationRemarks: 90; verbatimLatitude: 40°26'2.76"N; verbatimLongitude: 26°33'22.85"E; **Identification:** identificationID: esoguent-th-ıd-83; **Event:** eventDate: 06/05/2002
- **Type status:** Other material. **Occurrence:** sex: 2 males pharate adult, 2 females pharate adult; lifeStage: 6 pupae, 8 larvae; **Taxon:** scientificName: Simulium (Eusimulium) velutinum (Santos Abreu, 1922); kingdom: Animalia; phylum: Arthropoda; class: Insecta; order: Diptera; family: Simuliidae; genus: Simulium; subgenus: Eusimulium; scientificNameAuthorship: (Santos Abreu, 1922); **Location:** continent: Europe; country: Turkey; stateProvince: Çanakkale; county: Gelibolu; locationRemarks: 91; verbatimLatitude: 40°24'36.21"N; verbatimLongitude: 26°30'21.36"E; **Identification:** identificationID: esoguent-th-ıd-84; **Event:** eventDate: 06/05/2002
- **Type status:** Other material. **Occurrence:** sex: 1 male pharate adult; lifeStage: 6 pupae, 9 larvae; **Taxon:** scientificName: Simulium (Eusimulium) velutinum (Santos Abreu, 1922); kingdom: Animalia; phylum: Arthropoda; class: Insecta; order: Diptera; family: Simuliidae; genus: Simulium; subgenus: Eusimulium; scientificNameAuthorship: (Santos Abreu, 1922); **Location:** continent: Europe; country: Turkey; stateProvince: Çanakkale; county: Gelibolu; locationRemarks: 92; verbatimLatitude: 40°22'47.18"N; verbatimLongitude: 26°27'34.33"E; **Identification:** identificationID: esoguent-th-ıd-85; **Event:** eventDate: 06/05/2002
- **Type status:** Other material. **Occurrence:** sex: 2 males pharate adult; lifeStage: 5 pupae, 3 larvae; **Taxon:** scientificName: Simulium (Eusimulium) velutinum (Santos Abreu, 1922); kingdom: Animalia; phylum: Arthropoda; class: Insecta; order: Diptera; family: Simuliidae; genus: Simulium; subgenus: Eusimulium; scientificNameAuthorship: (Santos Abreu, 1922); **Location:** continent: Europe; country: Turkey; stateProvince: Tekirdağ; county: Kumbağ; locationRemarks: 108; verbatimLatitude: 40°47'52.99"N; verbatimLongitude: 27°21'48.85"E; **Identification:** identificationID: esoguent-th-ıd-86; **Event:** eventDate: 06/08/2002

#### Notes

*Simulium velutinum* occurs in most of Europe but is especially common in the Mediterranean subregion from the Canary Islands (type locality) to Israel and Jordan (Crosskey and Malicky 2001). According to Kazancı and Ertunç (2008)​, *S. velutinum* also inhabits southwestern Anatolia. Its existence in Turkish Thrace is, therefore, expected. Identification of this species is most reliably based on genitalia: the ventral plate of the male is smaller and more triangular and the spermatheca of the female is without the usual sclerotized nipple-like extension that is found in the other members of the *S. aureum* group.

### Simulium (Nevermannia) cryophilum complex

#### Materials

- **Type status:** Other material. **Occurrence:** lifeStage: 4 pupae, 6 larvae; **Taxon:** scientificName: Simulium (Nevermannia) cryophilum complex; kingdom: Animalia; phylum: Arthropoda; class: Insecta; order: Diptera; family: Simuliidae; genus: Simulium; subgenus: Nevermannia; **Location:** continent: Europe; country: Turkey; stateProvince: Tekirdağ; county: Kumbağ; locationRemarks: 106; verbatimLatitude: 40°49'39.01"N; verbatimLongitude: 27°23'8.57"E; **Identification:** identificationID: esoguent-th-ıd-87; **Event:** eventDate: 06/08/2002
- **Type status:** Other material. **Occurrence:** lifeStage: 3 pupae, 5 larvae; **Taxon:** scientificName: Simulium (Nevermannia) cryophilum complex; kingdom: Animalia; phylum: Arthropoda; class: Insecta; order: Diptera; family: Simuliidae; genus: Simulium; subgenus: Nevermannia; **Location:** continent: Europe; country: Turkey; stateProvince: Tekirdağ; county: Kumbağ; locationRemarks: 108; verbatimLatitude: 40°47'52.99"N; verbatimLongitude: 27°21'48.85"E; **Identification:** identificationID: esoguent-th-ıd-88; **Event:** eventDate: 06/08/2002

#### Notes

*Simulium cryophilum* is a member of the *S. vernum* species-group, which includes more than one-fourth of the nominal species in the western Palearctic. According to Crosskey and Malicky (2001), there are difficulties in identification of the species of the *S. vernum* group. Another taxonomic difficulty is that *S. cryophilum* is a composite of at least two chromosomally distinct sibling species (Hunter 1987; Adler et al. 1999). We, therefore, refer to it in Turkey as the *S. cryophilum* complex. We found it in two streams in the southern part of our study region. The main structural features of our material were in the pupal stage: (1) gill with all four filaments held closely together and about equal in thickness (Bass 1998; Crosskey and Malicky 2001); (2) upper pair of gill filaments branched horizontally (Jedlićka et al. 2004); (3) upper and lower pairs of filaments on short common stalks equal in length (Bass 1998; Jedlićka et al. 2004); (4) thoracic microtubercles dense and rounded and thoracic trichomes simple (Bass 1998); and (5) cocoon fully covering pupa and with a short anteromedian horn. In our larvae, the postgenal cleft was clearly defined and pentagonal, conforming to the description by Bass (1998)​.

### Simulium (Simulium) bezzii

(Corti, 1914)

#### Materials

- **Type status:** Other material. **Occurrence:** lifeStage: 2 pupae; **Taxon:** scientificName: Simulium (Simulium) bezzii (Corti, 1914); kingdom: Animalia; phylum: Arthropoda; class: Insecta; order: Diptera; family: Simuliidae; genus: Simulium; subgenus: Simulium; scientificNameAuthorship: (Corti, 1914); **Location:** continent: Europe; country: Turkey; stateProvince: Kırklareli; county: Demirköy; locationRemarks: 35; verbatimLatitude: 41°52'21.65"N; verbatimLongitude: 27°54'3.96"E; **Identification:** identificationID: esoguent-th-ıd-89; **Event:** eventDate: 05/26/2002
- **Type status:** Other material. **Occurrence:** lifeStage: 4 pupae, 1 larvae; **Taxon:** scientificName: Simulium (Simulium) bezzii (Corti, 1914); kingdom: Animalia; phylum: Arthropoda; class: Insecta; order: Diptera; family: Simuliidae; genus: Simulium; subgenus: Simulium; scientificNameAuthorship: (Corti, 1914); **Location:** continent: Europe; country: Turkey; stateProvince: Kırklareli; county: İğneada; locationRemarks: 36; verbatimLatitude: 41°49'16.69"N; verbatimLongitude: 27°57'8.53"E; **Identification:** identificationID: esoguent-th-ıd-90; **Event:** eventDate: 05/26/2002
- **Type status:** Other material. **Occurrence:** lifeStage: 5 pupae, 3 larvae; **Taxon:** scientificName: Simulium (Simulium) bezzii (Corti, 1914); kingdom: Animalia; phylum: Arthropoda; class: Insecta; order: Diptera; family: Simuliidae; genus: Simulium; subgenus: Simulium; scientificNameAuthorship: (Corti, 1914); **Location:** continent: Europe; country: Turkey; stateProvince: Kırklareli; county: Kofçaz; locationRemarks: 47; verbatimLatitude: 41°52'37.87"N; verbatimLongitude: 27°10'41.95"E; **Identification:** identificationID: esoguent-th-ıd-91; **Event:** eventDate: 05/28/2002
- **Type status:** Other material. **Occurrence:** sex: 2 females; lifeStage: 3 pupae, 2 larvae; **Taxon:** scientificName: Simulium (Simulium) bezzii (Corti, 1914); kingdom: Animalia; phylum: Arthropoda; class: Insecta; order: Diptera; family: Simuliidae; genus: Simulium; subgenus: Simulium; scientificNameAuthorship: (Corti, 1914); **Location:** continent: Europe; country: Turkey; stateProvince: Kırklareli; county: Kofçaz; locationRemarks: 48; verbatimLatitude: 41°54'8.88"N; verbatimLongitude: 27°10'30.90"E; **Identification:** identificationID: esoguent-th-ıd-92; **Event:** eventDate: 05/28/2002
- **Type status:** Other material. **Occurrence:** lifeStage: 6 pupae, 1 larvae; **Taxon:** scientificName: Simulium (Simulium) bezzii (Corti, 1914); kingdom: Animalia; phylum: Arthropoda; class: Insecta; order: Diptera; family: Simuliidae; genus: Simulium; subgenus: Simulium; scientificNameAuthorship: (Corti, 1914); **Location:** continent: Europe; country: Turkey; stateProvince: Kırklareli; county: Kofçaz; locationRemarks: 49; verbatimLatitude: 41°55'33.95"N; verbatimLongitude: 27°9'48.15"E; **Identification:** identificationID: esoguent-th-ıd-93; **Event:** eventDate: 05/28/2002
- **Type status:** Other material. **Occurrence:** lifeStage: 4 pupae, 3 larvae; **Taxon:** scientificName: Simulium (Simulium) bezzii (Corti, 1914); kingdom: Animalia; phylum: Arthropoda; class: Insecta; order: Diptera; family: Simuliidae; genus: Simulium; subgenus: Simulium; scientificNameAuthorship: (Corti, 1914); **Location:** continent: Europe; country: Turkey; stateProvince: Kırklareli; county: Kofçaz; locationRemarks: 50; verbatimLatitude: 41°56'53.66"N; verbatimLongitude: 27°9'42.23"E; **Identification:** identificationID: esoguent-th-ıd-94; **Event:** eventDate: 05/28/2002
- **Type status:** Other material. **Occurrence:** lifeStage: 2 pupae, 2 larvae; **Taxon:** scientificName: Simulium (Simulium) bezzii (Corti, 1914); kingdom: Animalia; phylum: Arthropoda; class: Insecta; order: Diptera; family: Simuliidae; genus: Simulium; subgenus: Simulium; scientificNameAuthorship: (Corti, 1914); **Location:** continent: Europe; country: Turkey; stateProvince: Kırklareli; county: Kofçaz; locationRemarks: 51; verbatimLatitude: 41°56'22.44"N; verbatimLongitude: 27°5'33.09"E; **Identification:** identificationID: esoguent-th-ıd-95; **Event:** eventDate: 05/28/2002
- **Type status:** Other material. **Occurrence:** lifeStage: 6 pupae; **Taxon:** scientificName: Simulium (Simulium) bezzii (Corti, 1914); kingdom: Animalia; phylum: Arthropoda; class: Insecta; order: Diptera; family: Simuliidae; genus: Simulium; subgenus: Simulium; scientificNameAuthorship: (Corti, 1914); **Location:** continent: Europe; country: Turkey; stateProvince: Kırklareli; county: Kofçaz; locationRemarks: 52; verbatimLatitude: 41°56'50.96"N; verbatimLongitude: 27°3'41.00"E; **Identification:** identificationID: esoguent-th-ıd-96; **Event:** eventDate: 05/28/2002
- **Type status:** Other material. **Occurrence:** lifeStage: 4 pupae, 2 larvae; **Taxon:** scientificName: Simulium (Simulium) bezzii (Corti, 1914); kingdom: Animalia; phylum: Arthropoda; class: Insecta; order: Diptera; family: Simuliidae; genus: Simulium; subgenus: Simulium; scientificNameAuthorship: (Corti, 1914); **Location:** continent: Europe; country: Turkey; stateProvince: Edirne; county: Lalapaşa; locationRemarks: 53; verbatimLatitude: 41°58'26.47"N; verbatimLongitude: 27°0'28.78"E; **Identification:** identificationID: esoguent-th-ıd-97; **Event:** eventDate: 05/28/2002
- **Type status:** Other material. **Occurrence:** lifeStage: 5 pupae, 2 larvae; **Taxon:** scientificName: Simulium (Simulium) bezzii (Corti, 1914); kingdom: Animalia; phylum: Arthropoda; class: Insecta; order: Diptera; family: Simuliidae; genus: Simulium; subgenus: Simulium; scientificNameAuthorship: (Corti, 1914); **Location:** continent: Europe; country: Turkey; stateProvince: Edirne; county: Keşan; locationRemarks: 80; verbatimLatitude: 40°43'36.00"N; verbatimLongitude: 26°42'46.33"E; **Identification:** identificationID: esoguent-th-ıd-98; **Event:** eventDate: 06/03/2002
- **Type status:** Other material. **Occurrence:** sex: 1 male; lifeStage: 3 pupae / 1 pupae; **Taxon:** scientificName: Simulium (Simulium) bezzii (Corti, 1914); kingdom: Animalia; phylum: Arthropoda; class: Insecta; order: Diptera; family: Simuliidae; genus: Simulium; subgenus: Simulium; scientificNameAuthorship: (Corti, 1914); **Location:** continent: Europe; country: Turkey; stateProvince: Tekirdağ; county: Şarköy; locationRemarks: 82; verbatimLatitude: 40°41'28.95"N; verbatimLongitude: 27°5'45.01"E; **Identification:** identificationID: esoguent-th-ıd-99; **Event:** eventDate: 03.06.2002 / 05.07.2003
- **Type status:** Other material. **Occurrence:** lifeStage: 5 pupae, 1 larvae; **Taxon:** scientificName: Simulium (Simulium) bezzii (Corti, 1914); kingdom: Animalia; phylum: Arthropoda; class: Insecta; order: Diptera; family: Simuliidae; genus: Simulium; subgenus: Simulium; scientificNameAuthorship: (Corti, 1914); **Location:** continent: Europe; country: Turkey; stateProvince: Çanakkale; county: Gelibolu; locationRemarks: 91; verbatimLatitude: 40°24'36.21"N; verbatimLongitude: 26°30'21.36"E; **Identification:** identificationID: esoguent-th-ıd-100; **Event:** eventDate: 06/05/2002

#### Notes

This species occurs in the southwestern Palearctic from Iberia through central and southern Europe to the Transcaucasus and northern Middle East, and is present in northwestern Africa and most of the larger Mediterranean islands. It is common and widespread in Anatolia (Crosskey and Zwick 2007). *Simulium bezzii*, like *Simulium kerisorum*, also known from Turkey, has six pupal gill filaments, in contrast to most other species of the *S. bezzii*-group, which generally have eight filaments. It can be distinguished by large fenestrations posterior to the cocoon rim. Crosskey and Zwick (2007) stated that *S. kerisorum* is not a valid species and thought that the Turkish record of Kazancı and Clergue-Gazeau (1990) is questionable. The chromosomes of *Simulium bezzii* have been studied (mainly by Kachvoryan (1988), as *Tetisimulium condici*) but not those of *S. kerisorum*, and cytological comparison of the two nominal taxa is desirable (Crosskey and Zwick 2007​).

### Simulium (Simulium) bukovskii

Rubtsov, 1940

#### Materials

- **Type status:** Other material. **Occurrence:** lifeStage: 8 pupae; **Taxon:** scientificName: Simulium (Simulium) bukovskii Rubtsov, 1940; kingdom: Animalia; phylum: Arthropoda; class: Insecta; order: Diptera; family: Simuliidae; genus: Simulium; subgenus: Simulium; scientificNameAuthorship: Rubtsov, 1940; **Location:** continent: Europe; country: Turkey; stateProvince: Kırklareli; county: İğneada; locationRemarks: 36; verbatimLatitude: 41°49'16.69"N; verbatimLongitude: 27°57'8.53"E; **Identification:** identificationID: esoguent-th-ıd-101; **Event:** eventDate: 04/22/2005

#### Notes

The type locality of *Simulium bukovskii* is in Crimea (Ukraine) and its distribution ranges from the Balkans to Armenia. It was recorded from Turkey by different authors (Crosskey and Zwick 2007). Furthermore, it is similar to *S. degrangei* Dorier and Grenier (1960), which is also common in most countries of southern Europe. Crosskey and Zwick (2007)​ emphasized the similarity of the two species and that synonymy should be considered.

### Simulium (Simulium) noelleri

Friederichs, 1920

#### Materials

- **Type status:** Other material. **Occurrence:** lifeStage: 6 pupae, 9 larvae; **Taxon:** scientificName: Simulium (Simulium) noelleri Friederichs, 1920; kingdom: Animalia; phylum: Arthropoda; class: Insecta; order: Diptera; family: Simuliidae; genus: Simulium; subgenus: Simulium; scientificNameAuthorship: Friederichs, 1920; **Location:** continent: Europe; country: Turkey; stateProvince: Edirne; county: Meriç; locationRemarks: 71; verbatimLatitude: 41°7'27.26"N; verbatimLongitude: 26°25'32.17"E; **Identification:** identificationID: esoguent-th-ıd-102; **Event:** eventDate: 06/01/2002

#### Notes

Simulium (Simulium) noelleri is a well-known simuliid, with a general preference for outlets. It is distinguished by the branching arrangement of the pupal gills (3+3+2 or 3+2+2+1). We encountered it at one locality, an outlet from a small pond. Our pupae have eight gill filaments arranged as 3+3+2, and the apotome pattern of the larval head capsule is like an ill-defined “H”, as described by Bass (1998). Although, *S. noelleri* is widespread in the Palaearctic, there are rwo records of it in Turkey, from the Sakarya River basin (Sirin and Sahin 2005) and East Marmara Region (Şirin et al. 2014​).

### Simulium (Simulium) kiritshenkoi

Rubtsov, 1940

#### Materials

- **Type status:** Other material. **Occurrence:** lifeStage: 8 pupae, 12 larvae; **Taxon:** scientificName: Simulium (Simulium) kiritshenkoi Rubtsov, 1940; kingdom: Animalia; phylum: Arthropoda; class: Insecta; order: Diptera; family: Simuliidae; genus: Simulium; subgenus: Simulium; scientificNameAuthorship: Rubtsov, 1940; **Location:** continent: Europe; country: Turkey; stateProvince: İstanbul; county: Gaziosmanpaşa; locationRemarks: 1; verbatimLatitude: 41°11'6.44"N; verbatimLongitude: 28°58'58.40"E; **Identification:** identificationID: esoguent-th-ıd-103; **Event:** eventDate: 05/20/2002
- **Type status:** Other material. **Occurrence:** lifeStage: 6 pupae, 7 larvae; **Taxon:** scientificName: Simulium (Simulium) kiritshenkoi Rubtsov, 1940; kingdom: Animalia; phylum: Arthropoda; class: Insecta; order: Diptera; family: Simuliidae; genus: Simulium; subgenus: Simulium; scientificNameAuthorship: Rubtsov, 1940; **Location:** continent: Europe; country: Turkey; stateProvince: İstanbul; county: Gaziosmanpaşa; locationRemarks: 2; verbatimLatitude: 41°11'40.47"N; verbatimLongitude: 28°54'51.58"E; **Identification:** identificationID: esoguent-th-ıd-104; **Event:** eventDate: 05/19/2002
- **Type status:** Other material. **Occurrence:** lifeStage: 3 pupae; **Taxon:** scientificName: Simulium (Simulium) kiritshenkoi Rubtsov, 1940; kingdom: Animalia; phylum: Arthropoda; class: Insecta; order: Diptera; family: Simuliidae; genus: Simulium; subgenus: Simulium; scientificNameAuthorship: Rubtsov, 1940; **Location:** continent: Europe; country: Turkey; stateProvince: İstanbul; county: Çatalca; locationRemarks: 9; verbatimLatitude: 41°11'4.58"N; verbatimLongitude: 28°38'17.71"E; **Identification:** identificationID: esoguent-th-ıd-105; **Event:** eventDate: 05/22/2002
- **Type status:** Other material. **Occurrence:** lifeStage: 10 pupae, 11 larvae / 2 pupae; **Taxon:** scientificName: Simulium (Simulium) kiritshenkoi Rubtsov, 1940; kingdom: Animalia; phylum: Arthropoda; class: Insecta; order: Diptera; family: Simuliidae; genus: Simulium; subgenus: Simulium; scientificNameAuthorship: Rubtsov, 1940; **Location:** continent: Europe; country: Turkey; stateProvince: İstanbul; county: Çatalca; locationRemarks: 12; verbatimLatitude: 41°13'3.97"N; verbatimLongitude: 28°21'41.16"E; **Identification:** identificationID: esoguent-th-ıd-106; **Event:** eventDate: 22.05.2002 / 22.04.2005
- **Type status:** Other material. **Occurrence:** lifeStage: 9 pupae, 4 larvae; **Taxon:** scientificName: Simulium (Simulium) kiritshenkoi Rubtsov, 1940; kingdom: Animalia; phylum: Arthropoda; class: Insecta; order: Diptera; family: Simuliidae; genus: Simulium; subgenus: Simulium; scientificNameAuthorship: Rubtsov, 1940; **Location:** continent: Europe; country: Turkey; stateProvince: Kırklareli; county: Kıyıköy; locationRemarks: 26; verbatimLatitude: 41°38'59.36"N; verbatimLongitude: 27°58'2.28"E; **Identification:** identificationID: esoguent-th-ıd-107; **Event:** eventDate: 05/24/2002
- **Type status:** Other material. **Occurrence:** lifeStage: 6 pupae, 7 larvae; **Taxon:** scientificName: Simulium (Simulium) kiritshenkoi Rubtsov, 1940; kingdom: Animalia; phylum: Arthropoda; class: Insecta; order: Diptera; family: Simuliidae; genus: Simulium; subgenus: Simulium; scientificNameAuthorship: Rubtsov, 1940; **Location:** continent: Europe; country: Turkey; stateProvince: Kırklareli; county: Kıyıköy; locationRemarks: 27; verbatimLatitude: 41°37'33.39"N; verbatimLongitude: 27°53'35.61"E; **Identification:** identificationID: esoguent-th-ıd-108; **Event:** eventDate: 05/24/2002
- **Type status:** Other material. **Occurrence:** lifeStage: 4 pupae, 3 larvae; **Taxon:** scientificName: Simulium (Simulium) kiritshenkoi Rubtsov, 1940; kingdom: Animalia; phylum: Arthropoda; class: Insecta; order: Diptera; family: Simuliidae; genus: Simulium; subgenus: Simulium; scientificNameAuthorship: Rubtsov, 1940; **Location:** continent: Europe; country: Turkey; stateProvince: Kırklareli; county: Vize; locationRemarks: 28; verbatimLatitude: 41°36'22.59"N; verbatimLongitude: 27°42'49.15"E; **Identification:** identificationID: esoguent-th-ıd-109; **Event:** eventDate: 05/25/2002
- **Type status:** Other material. **Occurrence:** lifeStage: 4 pupae, 8 larvae; **Taxon:** scientificName: Simulium (Simulium) kiritshenkoi Rubtsov, 1940; kingdom: Animalia; phylum: Arthropoda; class: Insecta; order: Diptera; family: Simuliidae; genus: Simulium; subgenus: Simulium; scientificNameAuthorship: Rubtsov, 1940; **Location:** continent: Europe; country: Turkey; stateProvince: Kırklareli; county: Demirköy; locationRemarks: 34; verbatimLatitude: 41°51'42.45"N; verbatimLongitude: 27°48'14.71"E; **Identification:** identificationID: esoguent-th-ıd-110; **Event:** eventDate: 05/26/2002
- **Type status:** Other material. **Occurrence:** lifeStage: 6 pupae, 7 larvae; **Taxon:** scientificName: Simulium (Simulium) kiritshenkoi Rubtsov, 1940; kingdom: Animalia; phylum: Arthropoda; class: Insecta; order: Diptera; family: Simuliidae; genus: Simulium; subgenus: Simulium; scientificNameAuthorship: Rubtsov, 1940; **Location:** continent: Europe; country: Turkey; stateProvince: Kırklareli; county: Armağan; locationRemarks: 42; verbatimLatitude: 41°51'2.67"N; verbatimLongitude: 27°26'27.85"E; **Identification:** identificationID: esoguent-th-ıd-111; **Event:** eventDate: 05/27/2002
- **Type status:** Other material. **Occurrence:** lifeStage: 6 pupae, 4 larvae; **Taxon:** scientificName: Simulium (Simulium) kiritshenkoi Rubtsov, 1940; kingdom: Animalia; phylum: Arthropoda; class: Insecta; order: Diptera; family: Simuliidae; genus: Simulium; subgenus: Simulium; scientificNameAuthorship: Rubtsov, 1940; **Location:** continent: Europe; country: Turkey; stateProvince: Kırklareli; county: Dereköy; locationRemarks: 44; verbatimLatitude: 41°51'1.19"N; verbatimLongitude: 27°19'29.05"E; **Identification:** identificationID: esoguent-th-ıd-112; **Event:** eventDate: 05/27/2002
- **Type status:** Other material. **Occurrence:** lifeStage: 3 pupae, 3 larvae; **Taxon:** scientificName: Simulium (Simulium) kiritshenkoi Rubtsov, 1940; kingdom: Animalia; phylum: Arthropoda; class: Insecta; order: Diptera; family: Simuliidae; genus: Simulium; subgenus: Simulium; scientificNameAuthorship: Rubtsov, 1940; **Location:** continent: Europe; country: Turkey; stateProvince: Kırklareli; county: Merkez; locationRemarks: 45; verbatimLatitude: 41°47'2.65"N; verbatimLongitude: 27°12'27.76"E; **Identification:** identificationID: esoguent-th-ıd-113; **Event:** eventDate: 05/28/2002
- **Type status:** Other material. **Occurrence:** lifeStage: 9 pupae, 7 larvae; **Taxon:** scientificName: Simulium (Simulium) kiritshenkoi Rubtsov, 1940; kingdom: Animalia; phylum: Arthropoda; class: Insecta; order: Diptera; family: Simuliidae; genus: Simulium; subgenus: Simulium; scientificNameAuthorship: Rubtsov, 1940; **Location:** continent: Europe; country: Turkey; stateProvince: Kırklareli; county: Kofçaz; locationRemarks: 50; verbatimLatitude: 41°56'53.66"N; verbatimLongitude: 27°9'42.23"E; **Identification:** identificationID: esoguent-th-ıd-114; **Event:** eventDate: 05/28/2002
- **Type status:** Other material. **Occurrence:** lifeStage: 6 pupae, 9 larvae; **Taxon:** scientificName: Simulium (Simulium) kiritshenkoi Rubtsov, 1940; kingdom: Animalia; phylum: Arthropoda; class: Insecta; order: Diptera; family: Simuliidae; genus: Simulium; subgenus: Simulium; scientificNameAuthorship: Rubtsov, 1940; **Location:** continent: Europe; country: Turkey; stateProvince: Edirne; county: Merkez; locationRemarks: 57; verbatimLatitude: 41°44'12.71"N; verbatimLongitude: 26°39'26.00"E; **Identification:** identificationID: esoguent-th-ıd-115; **Event:** eventDate: 05/29/2002
- **Type status:** Other material. **Occurrence:** lifeStage: 8 pupae; **Taxon:** scientificName: Simulium (Simulium) kiritshenkoi Rubtsov, 1940; kingdom: Animalia; phylum: Arthropoda; class: Insecta; order: Diptera; family: Simuliidae; genus: Simulium; subgenus: Simulium; scientificNameAuthorship: Rubtsov, 1940; **Location:** continent: Europe; country: Turkey; stateProvince: Edirne; county: Hatipköy; locationRemarks: 62; verbatimLatitude: 41°47'40.09"N; verbatimLongitude: 26°34'10.89"E; **Identification:** identificationID: esoguent-th-ıd-116; **Event:** eventDate: 05/30/2002
- **Type status:** Other material. **Occurrence:** lifeStage: 4 pupae, 8 larvae; **Taxon:** scientificName: Simulium (Simulium) kiritshenkoi Rubtsov, 1940; kingdom: Animalia; phylum: Arthropoda; class: Insecta; order: Diptera; family: Simuliidae; genus: Simulium; subgenus: Simulium; scientificNameAuthorship: Rubtsov, 1940; **Location:** continent: Europe; country: Turkey; stateProvince: Edirne; county: Keşan; locationRemarks: 80; verbatimLatitude: 40°43'36.00"N; verbatimLongitude: 26°42'46.33"E; **Identification:** identificationID: esoguent-th-ıd-117; **Event:** eventDate: 06/03/2002
- **Type status:** Other material. **Occurrence:** lifeStage: 7 pupae, 4 larvae; **Taxon:** scientificName: Simulium (Simulium) kiritshenkoi Rubtsov, 1940; kingdom: Animalia; phylum: Arthropoda; class: Insecta; order: Diptera; family: Simuliidae; genus: Simulium; subgenus: Simulium; scientificNameAuthorship: Rubtsov, 1940; **Location:** continent: Europe; country: Turkey; stateProvince: Tekirdağ; county: Malkara; locationRemarks: 86; verbatimLatitude: 40°48'41.00"N; verbatimLongitude: 26°53'34.24"E; **Identification:** identificationID: esoguent-th-ıd-118; **Event:** eventDate: 06/04/2002
- **Type status:** Other material. **Occurrence:** lifeStage: 6 pupae, 5 larvae; **Taxon:** scientificName: Simulium (Simulium) kiritshenkoi Rubtsov, 1940; kingdom: Animalia; phylum: Arthropoda; class: Insecta; order: Diptera; family: Simuliidae; genus: Simulium; subgenus: Simulium; scientificNameAuthorship: Rubtsov, 1940; **Location:** continent: Europe; country: Turkey; stateProvince: Tekirdağ; county: Malkara; locationRemarks: 87; verbatimLatitude: 40°47'19.76"; verbatimLongitude: 26°48'15.30"E; **Identification:** identificationID: esoguent-th-ıd-119; **Event:** eventDate: 06/04/2002
- **Type status:** Other material. **Occurrence:** lifeStage: 7 pupae, 7 larvae; **Taxon:** scientificName: Simulium (Simulium) kiritshenkoi Rubtsov, 1940; kingdom: Animalia; phylum: Arthropoda; class: Insecta; order: Diptera; family: Simuliidae; genus: Simulium; subgenus: Simulium; scientificNameAuthorship: Rubtsov, 1940; **Location:** continent: Europe; country: Turkey; stateProvince: Çanakkale; county: Gelibolu; locationRemarks: 88; verbatimLatitude: 40°41'38.58"N; verbatimLongitude: 26°55'56.44"E; **Identification:** identificationID: esoguent-th-ıd-120; **Event:** eventDate: 06/04/2002
- **Type status:** Other material. **Occurrence:** lifeStage: 6 pupae, 5 larvae; **Taxon:** scientificName: Simulium (Simulium) kiritshenkoi Rubtsov, 1940; kingdom: Animalia; phylum: Arthropoda; class: Insecta; order: Diptera; family: Simuliidae; genus: Simulium; subgenus: Simulium; scientificNameAuthorship: Rubtsov, 1940; **Location:** continent: Europe; country: Turkey; stateProvince: Çanakkale; county: Gelibolu; locationRemarks: 89; verbatimLatitude: 40°42'4.87"N; verbatimLongitude: 26°54'53.59"E; **Identification:** identificationID: esoguent-th-ıd-121; **Event:** eventDate: 06/04/2002
- **Type status:** Other material. **Occurrence:** lifeStage: 6 pupae, 9 larvae; **Taxon:** scientificName: Simulium (Simulium) kiritshenkoi Rubtsov, 1940; kingdom: Animalia; phylum: Arthropoda; class: Insecta; order: Diptera; family: Simuliidae; genus: Simulium; subgenus: Simulium; scientificNameAuthorship: Rubtsov, 1940; **Location:** continent: Europe; country: Turkey; stateProvince: Tekirdağ; county: Malkara; locationRemarks: 95; verbatimLatitude: 40°58'38.79"N; verbatimLongitude: 26°49'16.96"E; **Identification:** identificationID: esoguent-th-ıd-122; **Event:** eventDate: 06/06/2002
- **Type status:** Other material. **Occurrence:** lifeStage: 7 pupae, 8 larvae; **Taxon:** scientificName: Simulium (Simulium) kiritshenkoi Rubtsov, 1940; kingdom: Animalia; phylum: Arthropoda; class: Insecta; order: Diptera; family: Simuliidae; genus: Simulium; subgenus: Simulium; scientificNameAuthorship: Rubtsov, 1940; **Location:** continent: Europe; country: Turkey; stateProvince: Kırklareli; county: Babaeski; locationRemarks: 104; verbatimLatitude: 41°25'18.74"N; verbatimLongitude: 27°10'47.04"E; **Identification:** identificationID: esoguent-th-ıd-123; **Event:** eventDate: 06/07/2002
- **Type status:** Other material. **Occurrence:** lifeStage: 2 pupae, 2 larvae; **Taxon:** scientificName: Simulium (Simulium) kiritshenkoi Rubtsov, 1940; kingdom: Animalia; phylum: Arthropoda; class: Insecta; order: Diptera; family: Simuliidae; genus: Simulium; subgenus: Simulium; scientificNameAuthorship: Rubtsov, 1940; **Location:** continent: Europe; country: Turkey; stateProvince: Tekirdağ; county: Şarköy; locationRemarks: 114; verbatimLatitude: 40°48'8.19"N; verbatimLongitude: 27°11'3.18"E; **Identification:** identificationID: esoguent-th-ıd-124; **Event:** eventDate: 06/09/2002
- **Type status:** Other material. **Occurrence:** lifeStage: 4 pupae, 5 larvae; **Taxon:** scientificName: Simulium (Simulium) kiritshenkoi Rubtsov, 1940; kingdom: Animalia; phylum: Arthropoda; class: Insecta; order: Diptera; family: Simuliidae; genus: Simulium; subgenus: Simulium; scientificNameAuthorship: Rubtsov, 1940; **Location:** continent: Europe; country: Turkey; stateProvince: Tekirdağ; county: Şarköy; locationRemarks: 115; verbatimLatitude: 40°47'10.21"N; verbatimLongitude: 27°9'40.88"E; **Identification:** identificationID: esoguent-th-ıd-125; **Event:** eventDate: 06/09/2002
- **Type status:** Other material. **Occurrence:** sex: 1 male; lifeStage: 7 pupae, 6 larvae; **Taxon:** scientificName: Simulium (Simulium) kiritshenkoi Rubtsov, 1940; kingdom: Animalia; phylum: Arthropoda; class: Insecta; order: Diptera; family: Simuliidae; genus: Simulium; subgenus: Simulium; scientificNameAuthorship: Rubtsov, 1940; **Location:** continent: Europe; country: Turkey; stateProvince: Tekirdağ; county: Malkara; locationRemarks: 117; verbatimLatitude: 40°55'58.80"N; verbatimLongitude: 27°12'7.01"E; **Identification:** identificationID: esoguent-th-ıd-126; **Event:** eventDate: 06/10/2002
- **Type status:** Other material. **Occurrence:** lifeStage: 8 pupae, 4 larvae; **Taxon:** scientificName: Simulium (Simulium) kiritshenkoi Rubtsov, 1940; kingdom: Animalia; phylum: Arthropoda; class: Insecta; order: Diptera; family: Simuliidae; genus: Simulium; subgenus: Simulium; scientificNameAuthorship: Rubtsov, 1940; **Location:** continent: Europe; country: Turkey; stateProvince: İstanbul; county: Silivri; locationRemarks: 122; verbatimLatitude: 41°13'57.53"N; verbatimLongitude: 28°7'5.05"E; **Identification:** identificationID: esoguent-th-ıd-127; **Event:** eventDate: 06/11/2002

#### Notes

This species is a member of the *S. ornatum* species-group, which presents some of the most difficult taxonomic problems among Palearctic simuliids, in part because of the descriptions of many nominal species. Until now, six species of this group (*baracorne* Smart, 1944, *fontanum* Terteryan, 1952, *kiritshenkoi* Rubtsov, 1940, *caucasicum* Rubtsov, 1940, *ornatum* Meigen, 1818 (complex), *trifasciatum* Curtis, 1839) have been recorded from Turkey by different authors, as well as *ornatum* s.l. Meigen reported from Thrace by Zwick (2007). Our material includes mainly aquatic stages, with only one reared adult, a male from Site 117. We identified this male as *S. kiritshenkoi* on the basis of the color of the antennae (orange-red) and legs (reddish yellow), as described by Crosskey (2002). The genitalia also conform to the description of Crosskey (2002): ventral plate beak-like process in profile narrow and slightly upturned, and dentate crest slightly overhanging the base of the beak-like process. We also have pupae and larvae from this locality and the taxonomic characters fit the description of *S. kiritshenkoi* by Rubtsov (1956), Terteryan (1968), Crosskey (2002) and Crosskey and Zwick (2007)​. The pupal characters are as follows: thoracic microtubercles dome-like and fairly sparse, cocoon slipper-shaped and with delicately to strongly perforated weave, gill filaments in four pairs on short common stems. The postgenal cleft of our larvae is comparatively small, subquadrate or slightly pentagonal and extended less than halfway toward the base of the hypostoma.

### Simulium (Simulium) reptans

(Linnaeus, 1758)

#### Materials

- **Type status:** Other material. **Occurrence:** lifeStage: 6 pupae, 5 larvae / 19 pupae, 5 larvae; **Taxon:** scientificName: Simulium (Simulium) reptans (Linnaeus, 1758); kingdom: Animalia; phylum: Arthropoda; class: Insecta; order: Diptera; family: Simuliidae; genus: Simulium; subgenus: Simulium; scientificNameAuthorship: (Linnaeus, 1758); **Location:** continent: Europe; country: Turkey; stateProvince: Kırklareli; county: İğneada; locationRemarks: 36; verbatimLatitude: 41°49'16.69"N; verbatimLongitude: 27°57'8.53"E; **Identification:** identificationID: esoguent-th-ıd-128; **Event:** eventDate: 26.05.2002 / 22.04.2005
- **Type status:** Other material. **Occurrence:** lifeStage: 7 pupae, 11 larvae / 1 pupae; **Taxon:** scientificName: Simulium (Simulium) reptans (Linnaeus, 1758); kingdom: Animalia; phylum: Arthropoda; class: Insecta; order: Diptera; family: Simuliidae; genus: Simulium; subgenus: Simulium; scientificNameAuthorship: (Linnaeus, 1758); **Location:** continent: Europe; country: Turkey; stateProvince: Kırklareli; county: Pınarhisar; locationRemarks: 38; verbatimLatitude: 41°40'11.14"N; verbatimLongitude: 27°26'32.75"E; **Identification:** identificationID: esoguent-th-ıd-129; **Event:** eventDate: 07/08/2003
- **Type status:** Other material. **Occurrence:** lifeStage: 5 pupae, 3 larvae; **Taxon:** scientificName: Simulium (Simulium) reptans (Linnaeus, 1758); kingdom: Animalia; phylum: Arthropoda; class: Insecta; order: Diptera; family: Simuliidae; genus: Simulium; subgenus: Simulium; scientificNameAuthorship: (Linnaeus, 1758); **Location:** continent: Europe; country: Turkey; stateProvince: Kırklareli; county: Merkez; locationRemarks: 46; verbatimLatitude: 41°49'48.43"N; verbatimLongitude: 27°10'42.01"E; **Identification:** identificationID: esoguent-th-ıd-130; **Event:** eventDate: 04/23/2005
- **Type status:** Other material. **Occurrence:** lifeStage: 8 pupae, 7 larvae; **Taxon:** scientificName: Simulium (Simulium) reptans (Linnaeus, 1758); kingdom: Animalia; phylum: Arthropoda; class: Insecta; order: Diptera; family: Simuliidae; genus: Simulium; subgenus: Simulium; scientificNameAuthorship: (Linnaeus, 1758); **Location:** continent: Europe; country: Turkey; stateProvince: Edirne; county: Lalapaşa; locationRemarks: 53; verbatimLatitude: 41°58'26.47"N; verbatimLongitude: 27°0'28.78"E; **Identification:** identificationID: esoguent-th-ıd-131; **Event:** eventDate: 05/28/2002
- **Type status:** Other material. **Occurrence:** lifeStage: 7 pupae, 8 larvae; **Taxon:** scientificName: Simulium (Simulium) reptans (Linnaeus, 1758); kingdom: Animalia; phylum: Arthropoda; class: Insecta; order: Diptera; family: Simuliidae; genus: Simulium; subgenus: Simulium; scientificNameAuthorship: (Linnaeus, 1758); **Location:** continent: Europe; country: Turkey; stateProvince: Edirne; county: Hatipköy; locationRemarks: 63; verbatimLatitude: 41°48'14.99"N; verbatimLongitude: 26°33'13.31"E; **Identification:** identificationID: esoguent-th-ıd-132; **Event:** eventDate: 05/30/2002

#### Notes

*Simulium reptans* is one of the most common black flies in the Palaearctic, distributed from China to Portugal and from Lapland to Italy. It is a human- and cattle-biting species (Day et al. 2008). In Turkey, this species were reported from the Büyük Menderes river basin in western Anatolia (Kazancı and Ertunç 2008ile and the va districts of thestanbul (Şirin et al. 2014). *Simulium reptans* is similar to *Simulium galeratum* Edwards, especially in the larval stages, and some authors (e.g. Crosskey and Howard 2004) regarded *galeratum* as a synonym of *reptans.* However, Day et al. (2008) and Bernotiene and Stunzėnas (2009) showed, by analyzing mitochondrial cytochrome *c* oxidase 1 gene sequences, that *S. galeratum* is a distinct species. According to Bass (1998), Knoz (1965) and Jedlićka et al. (2004), the most distinctive pupal character between *S. reptans* and *S. galeratum* is the conformation and distribution of microtubercles on the cuticle of the head and thorax; in *S. galeratum*, the microtubercles are sparse and pointed, but dense in *S. reptans*, they are dense and rounded. In our study, the pupae have dense, rounded microtubercles. However, the head capsule pigmentation of our larvae conforms to that of *S. reptans*, as given by Bass (1998)​. We did not observe variation among specimens from the four localities where we collected the species.

### Simulium (Simulium) variegatum

Meigen, 1818

#### Materials

- **Type status:** Other material. **Occurrence:** lifeStage: 5 pupae, 3 larvae / 2 pupae; **Taxon:** scientificName: Simulium (Simulium) variegatum Meigen, 1818; kingdom: Animalia; phylum: Arthropoda; class: Insecta; order: Diptera; family: Simuliidae; genus: Simulium; subgenus: Simulium; scientificNameAuthorship: Meigen, 1818; **Location:** continent: Europe; country: Turkey; stateProvince: Tekirdağ; county: Saray; locationRemarks: 23; verbatimLatitude: 41°28'49.33"N; verbatimLongitude: 27°57'11.84"E; **Identification:** identificationID: esoguent-th-ıd-133; **Event:** eventDate: 24.05.2002 / 09.07.2003
- **Type status:** Other material. **Occurrence:** lifeStage: 5 pupae, 6 larvae; **Taxon:** scientificName: Simulium (Simulium) variegatum Meigen, 1818; kingdom: Animalia; phylum: Arthropoda; class: Insecta; order: Diptera; family: Simuliidae; genus: Simulium; subgenus: Simulium; scientificNameAuthorship: Meigen, 1818; **Location:** continent: Europe; country: Turkey; stateProvince: Tekirdağ; county: Saray; locationRemarks: 24; verbatimLatitude: 41°32'57.72"N; verbatimLongitude: 28°2'32.53"E; **Identification:** identificationID: esoguent-th-ıd-134; **Event:** eventDate: 05/24/2002
- **Type status:** Other material. **Occurrence:** sex: 1 male; lifeStage: 9 pupae, 6 larvae; **Taxon:** scientificName: Simulium (Simulium) variegatum Meigen, 1818; kingdom: Animalia; phylum: Arthropoda; class: Insecta; order: Diptera; family: Simuliidae; genus: Simulium; subgenus: Simulium; scientificNameAuthorship: Meigen, 1818; **Location:** continent: Europe; country: Turkey; stateProvince: Tekirdağ; county: Saray; locationRemarks: 25; verbatimLatitude: 41°33'36.45"N; verbatimLongitude: 28°3'50.97"E; **Identification:** identificationID: esoguent-th-ıd-135; **Event:** eventDate: 05/24/2002
- **Type status:** Other material. **Occurrence:** lifeStage: 8 pupae, 7 larvae; **Taxon:** scientificName: Simulium (Simulium) variegatum Meigen, 1818; kingdom: Animalia; phylum: Arthropoda; class: Insecta; order: Diptera; family: Simuliidae; genus: Simulium; subgenus: Simulium; scientificNameAuthorship: Meigen, 1818; **Location:** continent: Europe; country: Turkey; stateProvince: Kırklareli; county: Kıyıköy; locationRemarks: 26; verbatimLatitude: 41°38'59.36"N; verbatimLongitude: 27°58'2.28"E; **Identification:** identificationID: esoguent-th-ıd-136; **Event:** eventDate: 05/24/2002
- **Type status:** Other material. **Occurrence:** lifeStage: 6 pupae, 4 larvae; **Taxon:** scientificName: Simulium (Simulium) variegatum Meigen, 1818; kingdom: Animalia; phylum: Arthropoda; class: Insecta; order: Diptera; family: Simuliidae; genus: Simulium; subgenus: Simulium; scientificNameAuthorship: Meigen, 1818; **Location:** continent: Europe; country: Turkey; stateProvince: Kırklareli; county: Pınarhisar; locationRemarks: 31; verbatimLatitude: 41°44'18.42"N; verbatimLongitude: 27°37'36.24"E; **Identification:** identificationID: esoguent-th-ıd-137; **Event:** eventDate: 05/25/2002
- **Type status:** Other material. **Occurrence:** lifeStage: 4 pupae, 7 larvae / 1 pupae; **Taxon:** scientificName: Simulium (Simulium) variegatum Meigen, 1818; kingdom: Animalia; phylum: Arthropoda; class: Insecta; order: Diptera; family: Simuliidae; genus: Simulium; subgenus: Simulium; scientificNameAuthorship: Meigen, 1818; **Location:** continent: Europe; country: Turkey; stateProvince: Kırklareli; county: Pınarhisar; locationRemarks: 32; verbatimLatitude: 41°46'52.34"N; verbatimLongitude: 27°42'18.82"E; **Identification:** identificationID: esoguent-th-ıd-138; **Event:** eventDate: 25.05.2002 / 08.07.2003
- **Type status:** Other material. **Occurrence:** lifeStage: 3 pupae, 1 larvae; **Taxon:** scientificName: Simulium (Simulium) variegatum Meigen, 1818; kingdom: Animalia; phylum: Arthropoda; class: Insecta; order: Diptera; family: Simuliidae; genus: Simulium; subgenus: Simulium; scientificNameAuthorship: Meigen, 1818; **Location:** continent: Europe; country: Turkey; stateProvince: Kırklareli; county: Üsküp; locationRemarks: 41; verbatimLatitude: 41°48'7.98"N; verbatimLongitude: 27°27'14.63"E; **Identification:** identificationID: esoguent-th-ıd-139; **Event:** eventDate: 05/27/2002
- **Type status:** Other material. **Occurrence:** sex: 1 female; lifeStage: 5 pupae, 3 larvae; **Taxon:** scientificName: Simulium (Simulium) variegatum Meigen, 1818; kingdom: Animalia; phylum: Arthropoda; class: Insecta; order: Diptera; family: Simuliidae; genus: Simulium; subgenus: Simulium; scientificNameAuthorship: Meigen, 1818; **Location:** continent: Europe; country: Turkey; stateProvince: Kırklareli; county: Kofçaz; locationRemarks: 52; verbatimLatitude: 41°56'50.96"N; verbatimLongitude: 27°3'41.00"E; **Identification:** identificationID: esoguent-th-ıd-140; **Event:** eventDate: 05/28/2002
- **Type status:** Other material. **Occurrence:** lifeStage: 6 pupae, 5 larvae; **Taxon:** scientificName: Simulium (Simulium) variegatum Meigen, 1818; kingdom: Animalia; phylum: Arthropoda; class: Insecta; order: Diptera; family: Simuliidae; genus: Simulium; subgenus: Simulium; scientificNameAuthorship: Meigen, 1818; **Location:** continent: Europe; country: Turkey; stateProvince: Tekirdağ; county: Kumbağ; locationRemarks: 106; verbatimLatitude: 40°49'39.01"N; verbatimLongitude: 27°23'8.57"E; **Identification:** identificationID: esoguent-th-ıd-141; **Event:** eventDate: 06/08/2002
- **Type status:** Other material. **Occurrence:** lifeStage: 4 pupae, 5 larvae; **Taxon:** scientificName: Simulium (Simulium) variegatum Meigen, 1818; kingdom: Animalia; phylum: Arthropoda; class: Insecta; order: Diptera; family: Simuliidae; genus: Simulium; subgenus: Simulium; scientificNameAuthorship: Meigen, 1818; **Location:** continent: Europe; country: Turkey; stateProvince: Tekirdağ; county: Kumbağ; locationRemarks: 107; verbatimLatitude: 40°48'27.93"N; verbatimLongitude: 27°23'8.49"E; **Identification:** identificationID: esoguent-th-ıd-142; **Event:** eventDate: 06/08/2002
- **Type status:** Other material. **Occurrence:** lifeStage: 8 pupae, 3 larvae; **Taxon:** scientificName: Simulium (Simulium) variegatum Meigen, 1818; kingdom: Animalia; phylum: Arthropoda; class: Insecta; order: Diptera; family: Simuliidae; genus: Simulium; subgenus: Simulium; scientificNameAuthorship: Meigen, 1818; **Location:** continent: Europe; country: Turkey; stateProvince: Tekirdağ; county: Kumbağ; locationRemarks: 108; verbatimLatitude: 40°47'52.99"N; verbatimLongitude: 27°21'48.85"E; **Identification:** identificationID: esoguent-th-ıd-143; **Event:** eventDate: 06/08/2002

#### Notes

The most diagnostic character of *Simulium variegatum* is the presence of two large thoracic bulges (patagia) near the pupal gill bases. We observed this feature in all specimens collected from 11 sites. This species is widely distributed in Turkey (Crosskey and Zwick 2007) and also common over most of Europe, western Asia and North Africa (Adler and Crosskey 2015​).

### Simulium (Simulium) argyreatum

Meigen, 1838

#### Materials

- **Type status:** Other material. **Occurrence:** lifeStage: 5 pupae, 4 larvae; **Taxon:** scientificName: Simulium (Simulium) argyreatum Meigen, 1838; kingdom: Animalia; phylum: Arthropoda; class: Insecta; order: Diptera; family: Simuliidae; genus: Simulium; subgenus: Simulium; scientificNameAuthorship: Meigen, 1838; **Location:** continent: Europe; country: Turkey; stateProvince: Kırklareli; county: Demirköy; locationRemarks: 33; verbatimLatitude: 41°49'22.89"N; verbatimLongitude: 27°45'48.04"E; **Identification:** identificationID: esoguent-th-ıd-144; **Event:** eventDate: 05/25/2002
- **Type status:** Other material. **Occurrence:** lifeStage: 5 pupae; **Taxon:** scientificName: Simulium (Simulium) argyreatum Meigen, 1838; kingdom: Animalia; phylum: Arthropoda; class: Insecta; order: Diptera; family: Simuliidae; genus: Simulium; subgenus: Simulium; scientificNameAuthorship: Meigen, 1838; **Location:** continent: Europe; country: Turkey; stateProvince: Kırklareli; county: Demirköy; locationRemarks: 37; verbatimLatitude: 41°48'24.50"N; verbatimLongitude: 27°49'21.60"E; **Identification:** identificationID: esoguent-th-ıd-145; **Event:** eventDate: 05/26/2002
- **Type status:** Other material. **Occurrence:** lifeStage: 9 pupae, 3 larvae; **Taxon:** scientificName: Simulium (Simulium) argyreatum Meigen, 1838; kingdom: Animalia; phylum: Arthropoda; class: Insecta; order: Diptera; family: Simuliidae; genus: Simulium; subgenus: Simulium; scientificNameAuthorship: Meigen, 1838; **Location:** continent: Europe; country: Turkey; stateProvince: Kırklareli; county: Dereköy; locationRemarks: 43; verbatimLatitude: 41°54'58.26"N; verbatimLongitude: 27°21'31.80"E; **Identification:** identificationID: esoguent-th-ıd-146; **Event:** eventDate: 05/27/2002
- **Type status:** Other material. **Occurrence:** lifeStage: 3 pupae, 2 larvae; **Taxon:** scientificName: Simulium (Simulium) argyreatum Meigen, 1838; kingdom: Animalia; phylum: Arthropoda; class: Insecta; order: Diptera; family: Simuliidae; genus: Simulium; subgenus: Simulium; scientificNameAuthorship: Meigen, 1838; **Location:** continent: Europe; country: Turkey; stateProvince: Edirne; county: Havsa; locationRemarks: 67; verbatimLatitude: 41°22'5.91"N; verbatimLongitude: 26°40'1.43"E; **Identification:** identificationID: esoguent-th-ıd-147; **Event:** eventDate: 06/01/2002

#### Notes

This species was reported from the Yedi Göller region in Bolu Province as a new record for Turkey (Kazancı and Ertunç 2008), and then recorded from Eastern Marmara Region (Şirin et al. 2014). It also is known from adjacent Balkan countries such as Bulgaria and Romania. We encountered the species at four localities, and identified it by pupal gill characters, using the keys of Knoz (1965), Jedlićka et al. (2004) and Yankovsky (2003)​: the filaments are directed downward at the base and then curve anteriorly; they lie close together proximally.

### Simulium (Trichodagmia) auricoma

Meigen, 1818

#### Materials

- **Type status:** Other material. **Occurrence:** lifeStage: 5 pupae, 2 larvae; **Taxon:** scientificName: Simulium (Trichodagmia) auricoma Meigen, 1818; kingdom: Animalia; phylum: Arthropoda; class: Insecta; order: Diptera; family: Simuliidae; genus: Simulium; subgenus: Trichodagmia; scientificNameAuthorship: Meigen, 1818; **Location:** continent: Europe; country: Turkey; stateProvince: Kırklareli; county: Kofçaz; locationRemarks: 52; verbatimLatitude: 41°56'50.96"N; verbatimLongitude: 27°3'41.00"E; **Identification:** identificationID: esoguent-th-ıd-148; **Event:** eventDate: 05/28/2002
- **Type status:** Other material. **Occurrence:** lifeStage: 3 pupae, 3 larvae; **Taxon:** scientificName: Simulium (Trichodagmia) auricoma Meigen, 1818; kingdom: Animalia; phylum: Arthropoda; class: Insecta; order: Diptera; family: Simuliidae; genus: Simulium; subgenus: Trichodagmia; scientificNameAuthorship: Meigen, 1818; **Location:** continent: Europe; country: Turkey; stateProvince: Çanakkale; county: Gelibolu; locationRemarks: 91; verbatimLatitude: 40°24'36.21"N; verbatimLongitude: 26°30'21.36"E; **Identification:** identificationID: esoguent-th-ıd-149; **Event:** eventDate: 06/05/2002

#### Notes

According to Crosskey and Zwick (2007), *Simulium auricoma* is spread through southern Europe from the Iberian to the Aegean islands, such as Lesbos and Rhodes, and occurs in mountainous areas. They reported it from Turkey for the first time and emphasized that it shows wide variation in the length, taper and spread of the filaments. Crosskey and Malicky (2001) noted that gill filaments vary from short, stubby, and minimally tapered, to long and gradually tapered; in all forms, the lowermost filament tends to diverge downward from the others. The filaments of our pupae from the two sites are short, stubby, and minimally tapered. The cocoon and genitalia characters of pharate males conform to the description by Crosskey and Malicky (2001)​.

<br/>

### Simulium (Wilhelmia) equinum

(Linnaeus, 1758)

#### Materials

- **Type status:** Other material. **Occurrence:** lifeStage: 5 pupae, 8 larvae; **Taxon:** scientificName: Simulium (Wilhelmia) equinum (Linnaeus, 1758); kingdom: Animalia; phylum: Arthropoda; class: Insecta; order: Diptera; family: Simuliidae; genus: Simulium; subgenus: Wilhelmia; scientificNameAuthorship: (Linnaeus, 1758); **Location:** continent: Europe; country: Turkey; stateProvince: İstanbul; county: Çatalca; locationRemarks: 16; verbatimLatitude: 41°23'13.96"N; verbatimLongitude: 28°22'1.34"E; **Identification:** identificationID: esoguent-th-ıd-150; **Event:** eventDate: 05/23/2002
- **Type status:** Other material. **Occurrence:** lifeStage: 2 pupae, 3 larvae; **Taxon:** scientificName: Simulium (Wilhelmia) equinum (Linnaeus, 1758); kingdom: Animalia; phylum: Arthropoda; class: Insecta; order: Diptera; family: Simuliidae; genus: Simulium; subgenus: Wilhelmia; scientificNameAuthorship: (Linnaeus, 1758); **Location:** continent: Europe; country: Turkey; stateProvince: İstanbul; county: Çatalca; locationRemarks: 17; verbatimLatitude: 41°23'54.45"N; verbatimLongitude: 28°19'8.26"E; **Identification:** identificationID: esoguent-th-ıd-151; **Event:** eventDate: 05/23/2002
- **Type status:** Other material. **Occurrence:** lifeStage: 4 pupae; **Taxon:** scientificName: Simulium (Wilhelmia) equinum (Linnaeus, 1758); kingdom: Animalia; phylum: Arthropoda; class: Insecta; order: Diptera; family: Simuliidae; genus: Simulium; subgenus: Wilhelmia; scientificNameAuthorship: (Linnaeus, 1758); **Location:** continent: Europe; country: Turkey; stateProvince: İstanbul; county: Çatalca; locationRemarks: 18; verbatimLatitude: 41°21'27.60"N; verbatimLongitude: 28°13'43.55"E; **Identification:** identificationID: esoguent-th-ıd-152; **Event:** eventDate: 05/23/2002
- **Type status:** Other material. **Occurrence:** sex: 2 rearing adults (1 male, 1 female); lifeStage: 7 pupae, 3 larvae; **Taxon:** scientificName: Simulium (Wilhelmia) equinum (Linnaeus, 1758); kingdom: Animalia; phylum: Arthropoda; class: Insecta; order: Diptera; family: Simuliidae; genus: Simulium; subgenus: Wilhelmia; scientificNameAuthorship: (Linnaeus, 1758); **Location:** continent: Europe; country: Turkey; stateProvince: İstanbul; county: Çatalca; locationRemarks: 19; verbatimLatitude: 41°23'5.91"N; verbatimLongitude: 28°11'54.84"E; **Identification:** identificationID: esoguent-th-ıd-153; **Event:** eventDate: 05/23/2002
- **Type status:** Other material. **Occurrence:** lifeStage: 5 pupae / 2 pupae; **Taxon:** scientificName: Simulium (Wilhelmia) equinum (Linnaeus, 1758); kingdom: Animalia; phylum: Arthropoda; class: Insecta; order: Diptera; family: Simuliidae; genus: Simulium; subgenus: Wilhelmia; scientificNameAuthorship: (Linnaeus, 1758); **Location:** continent: Europe; country: Turkey; stateProvince: İstanbul; county: Çatalca; locationRemarks: 20; verbatimLatitude: 41°24'2.09"N; verbatimLongitude: 28°10'3.80"E; **Identification:** identificationID: esoguent-th-ıd-154; **Event:** eventDate: 24.05.2002 / 09.07.2003
- **Type status:** Other material. **Occurrence:** lifeStage: 4 pupae, 10 larvae / 2 pupae; **Taxon:** scientificName: Simulium (Wilhelmia) equinum (Linnaeus, 1758); kingdom: Animalia; phylum: Arthropoda; class: Insecta; order: Diptera; family: Simuliidae; genus: Simulium; subgenus: Wilhelmia; scientificNameAuthorship: (Linnaeus, 1758); **Location:** continent: Europe; country: Turkey; stateProvince: Tekirdağ; county: Saray; locationRemarks: 23; verbatimLatitude: 41°28'49.33"N; verbatimLongitude: 27°57'11.84"E; **Identification:** identificationID: esoguent-th-ıd-155; **Event:** eventDate: 24.05.2002 / 09.07.2003
- **Type status:** Other material. **Occurrence:** lifeStage: 3 pupae, 7 larvae; **Taxon:** scientificName: Simulium (Wilhelmia) equinum (Linnaeus, 1758); kingdom: Animalia; phylum: Arthropoda; class: Insecta; order: Diptera; family: Simuliidae; genus: Simulium; subgenus: Wilhelmia; scientificNameAuthorship: (Linnaeus, 1758); **Location:** continent: Europe; country: Turkey; stateProvince: Edirne; county: İnece; locationRemarks: 55; verbatimLatitude: 41°40'13.00"N; verbatimLongitude: 27°4'7.90"E; **Identification:** identificationID: esoguent-th-ıd-156; **Event:** eventDate: 05/29/2002
- **Type status:** Other material. **Occurrence:** lifeStage: 2 pupae; **Taxon:** scientificName: Simulium (Wilhelmia) equinum (Linnaeus, 1758); kingdom: Animalia; phylum: Arthropoda; class: Insecta; order: Diptera; family: Simuliidae; genus: Simulium; subgenus: Wilhelmia; scientificNameAuthorship: (Linnaeus, 1758); **Location:** continent: Europe; country: Turkey; stateProvince: Edirne; county: İskender; locationRemarks: 65; verbatimLatitude: 41°35'21.77"N; verbatimLongitude: 26°40'57.69"E; **Identification:** identificationID: esoguent-th-ıd-157; **Event:** eventDate: 05/31/2002
- **Type status:** Other material. **Occurrence:** lifeStage: 4 pupae, 12 larvae; **Taxon:** scientificName: Simulium (Wilhelmia) equinum (Linnaeus, 1758); kingdom: Animalia; phylum: Arthropoda; class: Insecta; order: Diptera; family: Simuliidae; genus: Simulium; subgenus: Wilhelmia; scientificNameAuthorship: (Linnaeus, 1758); **Location:** continent: Europe; country: Turkey; stateProvince: Kırklareli; county: Babaeski; locationRemarks: 103; verbatimLatitude: 41°27'8.31"N; verbatimLongitude: 27°2'44.09"E; **Identification:** identificationID: esoguent-th-ıd-158; **Event:** eventDate: 06/07/2002

#### Notes

The pupae of *Simulium equinum* can be identified by the banana-like gill branches. Although *S. equinum* is found from central and northern Europe to northeastern Asia, it becomes progressively less common throughout the Mediterranean borderlands and islands (Crosskey and Zwick 2007). This species was reported from the Sakarya river system as a new record for Turkey (Sirin and Sahin 2005). Crosskey and Zwick (2007) also found it at a river near Kızılcahamam, Ankara. These authors note that *S. equinum* typically occurs with other species of the subgenus *Wilhelmia*. We record this species at nine sites, of which six were with *S. pseudequinum* and one with *S. balcanicum*.

### Simulium (Wilhelmia) balcanicum

(Enderlein, 1924)

#### Materials

- **Type status:** Other material. **Occurrence:** lifeStage: 14 pupae, 7 larvae; **Taxon:** scientificName: Simulium (Wilhelmia) balcanicum (Enderlein, 1924); kingdom: Animalia; phylum: Arthropoda; class: Insecta; order: Diptera; family: Simuliidae; genus: Simulium; subgenus: Wilhelmia; scientificNameAuthorship: (Enderlein, 1924); **Location:** continent: Europe; country: Turkey; stateProvince: Tekirdağ; county: Saray; locationRemarks: 25; verbatimLatitude: 41°33'36.45"N; verbatimLongitude: 28°3'50.97"E; **Identification:** identificationID: esoguent-th-ıd-159; **Event:** eventDate: 05/24/2002
- **Type status:** Other material. **Occurrence:** sex: 1 male pharate adult; lifeStage: 5 pupae, 9 larvae; **Taxon:** scientificName: Simulium (Wilhelmia) balcanicum (Enderlein, 1924); kingdom: Animalia; phylum: Arthropoda; class: Insecta; order: Diptera; family: Simuliidae; genus: Simulium; subgenus: Wilhelmia; scientificNameAuthorship: (Enderlein, 1924); **Location:** continent: Europe; country: Turkey; stateProvince: Edirne; county: Merkez; locationRemarks: 58; verbatimLatitude: 41°46'33.77"N; verbatimLongitude: 26°40'36.82"E; **Identification:** identificationID: esoguent-th-ıd-160; **Event:** eventDate: 05/29/2002
- **Type status:** Other material. **Occurrence:** sex: 3 males pharate adult; lifeStage: 8 pupae, 11 larvae; **Taxon:** scientificName: Simulium (Wilhelmia) balcanicum (Enderlein, 1924); kingdom: Animalia; phylum: Arthropoda; class: Insecta; order: Diptera; family: Simuliidae; genus: Simulium; subgenus: Wilhelmia; scientificNameAuthorship: (Enderlein, 1924); **Location:** continent: Europe; country: Turkey; stateProvince: Edirne; county: Merkez; locationRemarks: 61; verbatimLatitude: 41°43'25.74"N; verbatimLongitude: 26°33'38.19"E; **Identification:** identificationID: esoguent-th-ıd-161; **Event:** eventDate: 05/30/2002
- **Type status:** Other material. **Occurrence:** sex: 1 male; lifeStage: 6 pupae, 6 larvae; **Taxon:** scientificName: Simulium (Wilhelmia) balcanicum (Enderlein, 1924); kingdom: Animalia; phylum: Arthropoda; class: Insecta; order: Diptera; family: Simuliidae; genus: Simulium; subgenus: Wilhelmia; scientificNameAuthorship: (Enderlein, 1924); **Location:** continent: Europe; country: Turkey; stateProvince: Edirne; county: Hatipköy; locationRemarks: 63; verbatimLatitude: 41°48'14.99"N; verbatimLongitude: 26°33'13.31"E; **Identification:** identificationID: esoguent-th-ıd-162; **Event:** eventDate: 05/30/2002
- **Type status:** Other material. **Occurrence:** sex: 1 female pharate adult; lifeStage: 4 pupae, 6 larvae; **Taxon:** scientificName: Simulium (Wilhelmia) balcanicum (Enderlein, 1924); kingdom: Animalia; phylum: Arthropoda; class: Insecta; order: Diptera; family: Simuliidae; genus: Simulium; subgenus: Wilhelmia; scientificNameAuthorship: (Enderlein, 1924); **Location:** continent: Europe; country: Turkey; stateProvince: Edirne; county: Karaağaç; locationRemarks: 64; verbatimLatitude: 41°40'13.71"N; verbatimLongitude: 26°31'20.94"E; **Identification:** identificationID: esoguent-th-ıd-163; **Event:** eventDate: 05/31/2002
- **Type status:** Other material. **Occurrence:** lifeStage: 5 pupae, 9 larvae; **Taxon:** scientificName: Simulium (Wilhelmia) balcanicum (Enderlein, 1924); kingdom: Animalia; phylum: Arthropoda; class: Insecta; order: Diptera; family: Simuliidae; genus: Simulium; subgenus: Wilhelmia; scientificNameAuthorship: (Enderlein, 1924); **Location:** continent: Europe; country: Turkey; stateProvince: Edirne; county: Keşan; locationRemarks: 70; verbatimLatitude: 41°5'0.44"N; verbatimLongitude: 26°32'11.32"E; **Identification:** identificationID: esoguent-th-ıd-164; **Event:** eventDate: 06/01/2002
- **Type status:** Other material. **Occurrence:** lifeStage: 3 pupae, 5 larvae; **Taxon:** scientificName: Simulium (Wilhelmia) balcanicum (Enderlein, 1924); kingdom: Animalia; phylum: Arthropoda; class: Insecta; order: Diptera; family: Simuliidae; genus: Simulium; subgenus: Wilhelmia; scientificNameAuthorship: (Enderlein, 1924); **Location:** continent: Europe; country: Turkey; stateProvince: Edirne; county: Keşan; locationRemarks: 75; verbatimLatitude: 40°46'5.97"N; verbatimLongitude: 26°30'42.72"E; **Identification:** identificationID: esoguent-th-ıd-165; **Event:** eventDate: 06/02/2002
- **Type status:** Other material. **Occurrence:** sex: 1 female pharate adult; lifeStage: 2 pupae / 2 pupae; **Taxon:** scientificName: Simulium (Wilhelmia) balcanicum (Enderlein, 1924); kingdom: Animalia; phylum: Arthropoda; class: Insecta; order: Diptera; family: Simuliidae; genus: Simulium; subgenus: Wilhelmia; scientificNameAuthorship: (Enderlein, 1924); **Location:** continent: Europe; country: Turkey; stateProvince: Tekirdağ; county: Şarköy; locationRemarks: 83; verbatimLatitude: 40°46'21.52"N; verbatimLongitude: 27°3'32.09"E; **Identification:** identificationID: esoguent-th-ıd-166; **Event:** eventDate: 04.06.2002 / 05.07.2003
- **Type status:** Other material. **Occurrence:** sex: 1 male pharate adult; lifeStage: 1 pupa, 2 larvae; **Taxon:** scientificName: Simulium (Wilhelmia) balcanicum (Enderlein, 1924); kingdom: Animalia; phylum: Arthropoda; class: Insecta; order: Diptera; family: Simuliidae; genus: Simulium; subgenus: Wilhelmia; scientificNameAuthorship: (Enderlein, 1924); **Location:** continent: Europe; country: Turkey; stateProvince: Çanakkale; county: Gelibolu; locationRemarks: 90; verbatimLatitude: 40°26'2.76"N; verbatimLongitude: 26°33'22.85"E; **Identification:** identificationID: esoguent-th-ıd-167; **Event:** eventDate: 06/05/2002
- **Type status:** Other material. **Occurrence:** lifeStage: 3 pupae, 9 larvae; **Taxon:** scientificName: Simulium (Wilhelmia) balcanicum (Enderlein, 1924); kingdom: Animalia; phylum: Arthropoda; class: Insecta; order: Diptera; family: Simuliidae; genus: Simulium; subgenus: Wilhelmia; scientificNameAuthorship: (Enderlein, 1924); **Location:** continent: Europe; country: Turkey; stateProvince: Tekirdağ; county: Malkara; locationRemarks: 93; verbatimLatitude: 40°56'45.42"N; verbatimLongitude: 26°53'8.36"E; **Identification:** identificationID: esoguent-th-ıd-168; **Event:** eventDate: 06/06/2002
- **Type status:** Other material. **Occurrence:** lifeStage: 8 pupae; **Taxon:** scientificName: Simulium (Wilhelmia) balcanicum (Enderlein, 1924); kingdom: Animalia; phylum: Arthropoda; class: Insecta; order: Diptera; family: Simuliidae; genus: Simulium; subgenus: Wilhelmia; scientificNameAuthorship: (Enderlein, 1924); **Location:** continent: Europe; country: Turkey; stateProvince: Tekirdağ; county: Malkara; locationRemarks: 97; verbatimLatitude: 41°0'48.93"N; verbatimLongitude: 26°56'39.41"E; **Identification:** identificationID: esoguent-th-ıd-169; **Event:** eventDate: 06/06/2002
- **Type status:** Other material. **Occurrence:** lifeStage: 8 pupae, 4 larvae; **Taxon:** scientificName: Simulium (Wilhelmia) balcanicum (Enderlein, 1924); kingdom: Animalia; phylum: Arthropoda; class: Insecta; order: Diptera; family: Simuliidae; genus: Simulium; subgenus: Wilhelmia; scientificNameAuthorship: (Enderlein, 1924); **Location:** continent: Europe; country: Turkey; stateProvince: Tekirdağ; county: Hayrabolu; locationRemarks: 100; verbatimLatitude: 41°11'28.14"N; verbatimLongitude: 27°12'29.08"E; **Identification:** identificationID: esoguent-th-ıd-170; **Event:** eventDate: 06/06/2002
- **Type status:** Other material. **Occurrence:** sex: 1 male pharate adult; lifeStage: 3 pupae; **Taxon:** scientificName: Simulium (Wilhelmia) balcanicum (Enderlein, 1924); kingdom: Animalia; phylum: Arthropoda; class: Insecta; order: Diptera; family: Simuliidae; genus: Simulium; subgenus: Wilhelmia; scientificNameAuthorship: (Enderlein, 1924); **Location:** continent: Europe; country: Turkey; stateProvince: Kırklareli; county: Babaeski; locationRemarks: 103; verbatimLatitude: 41°27'8.31"N; verbatimLongitude: 27°2'44.09"E; **Identification:** identificationID: esoguent-th-ıd-171; **Event:** eventDate: 06/07/2002
- **Type status:** Other material. **Occurrence:** lifeStage: 2 pupae; **Taxon:** scientificName: Simulium (Wilhelmia) balcanicum (Enderlein, 1924); kingdom: Animalia; phylum: Arthropoda; class: Insecta; order: Diptera; family: Simuliidae; genus: Simulium; subgenus: Wilhelmia; scientificNameAuthorship: (Enderlein, 1924); **Location:** continent: Europe; country: Turkey; stateProvince: Kırklareli; county: Merkez; locationRemarks: 124; verbatimLatitude: 41°42'38.22"N; verbatimLongitude: 27°15'46.64"E; **Identification:** identificationID: esoguent-th-ıd-172; **Event:** eventDate: 07/08/2003
- **Type status:** Other material. **Occurrence:** lifeStage: 2 pupae; **Taxon:** scientificName: Simulium (Wilhelmia) balcanicum (Enderlein, 1924); kingdom: Animalia; phylum: Arthropoda; class: Insecta; order: Diptera; family: Simuliidae; genus: Simulium; subgenus: Wilhelmia; scientificNameAuthorship: (Enderlein, 1924); **Location:** continent: Europe; country: Turkey; stateProvince: Tekirdağ; county: Çorlu; locationRemarks: 130; verbatimLatitude: 41°12'41.18"N; verbatimLongitude: 27°57'15.98"E; **Identification:** identificationID: esoguent-th-ıd-173; **Event:** eventDate: 05/24/2006
- **Type status:** Other material. **Occurrence:** lifeStage: 5 pupae; **Taxon:** scientificName: Simulium (Wilhelmia) balcanicum (Enderlein, 1924); kingdom: Animalia; phylum: Arthropoda; class: Insecta; order: Diptera; family: Simuliidae; genus: Simulium; subgenus: Wilhelmia; scientificNameAuthorship: (Enderlein, 1924); **Location:** continent: Europe; country: Turkey; stateProvince: Tekirdağ; county: Çorlu; locationRemarks: 131; verbatimLatitude: 41°8'45.21"N; verbatimLongitude: 27°38'53.92"E; **Identification:** identificationID: esoguent-th-ıd-174; **Event:** eventDate: 05/24/2006

#### Notes

*Simulium balcanicum* was reported from four big river basins, Büyük Menderes, Yeşilırmak, Kızılırmak and Sakarya, in Anatolia according to the checklist of Turkish black flies (Kazancı and Ertunç 2008). Crosskey and Zwick (2007) stated that it is widespread in western Anatolia. Şirin et al. (2014) recorded this species eleven different streams in East Marmara Region. Additionally, *S. balcanicum* occurs in the adjacent western countries of Turkey, such as Bulgaria, Greece and Romania. *Simulium balcanicum* is similar to *S. lineatum*. The diagnostic character separating the two nominal species is in the pupal gill: in *S. lineatum* all six inner gill tubes arise independently from the gill base, whereas in *S. balcanicum* the posterior pair of inner gill tubes arises in the form of a fork with a common stem (Crosskey and Zwick 2007). All of our pupae and mature larvae have the *balcanicum* type of gills. Crosskey and Zwick (2007) doubted that *S. lineatum* and *S. balcanicum* are separate species. Because of similarity in the terminalia, the authors predicted that *S. balcanicum* is not a distinct species, and that the name probably should be synonymized with *S. lineatum*. They suggested, however, that chromosomal data for both nominal species need to be considered before establishing synonymy. Adler et al. (2012)​ analyzed the polytene chromosomes of these species in the Kızılırmak River and other sites in Europe and revealed that *S. lineatum* and *S. balcanicum* are distinct, reproductively isolated species. They stated that chromosomally, these two species have fixed-inversion differences and unique autosomal polymorphisms.

### Simulium (Wilhelmia) pseudequinum

Seguy, 1921

#### Materials

- **Type status:** Other material. **Occurrence:** lifeStage: 2 pupae; **Taxon:** scientificName: Simulium (Wilhelmia) pseudequinum Seguy, 1921; kingdom: Animalia; phylum: Arthropoda; class: Insecta; order: Diptera; family: Simuliidae; genus: Simulium; subgenus: Wilhelmia; scientificNameAuthorship: Seguy, 1921; **Location:** continent: Europe; country: Turkey; stateProvince: İstanbul; county: Kemerburgaz; locationRemarks: 3; verbatimLatitude: 41°9'48.99"N; verbatimLongitude: 28°54'53.02"E; **Identification:** identificationID: esoguent-th-ıd-175; **Event:** eventDate: 05/20/2002
- **Type status:** Other material. **Occurrence:** sex: 1 male, 1 female, 1 male pharate adult, 2 females pharate adult; lifeStage: 14 pupae, 13 larvae; **Taxon:** scientificName: Simulium (Wilhelmia) pseudequinum Seguy, 1921; kingdom: Animalia; phylum: Arthropoda; class: Insecta; order: Diptera; family: Simuliidae; genus: Simulium; subgenus: Wilhelmia; scientificNameAuthorship: Seguy, 1921; **Location:** continent: Europe; country: Turkey; stateProvince: İstanbul; county: Çatalca; locationRemarks: 12; verbatimLatitude: 41°13'3.97"N; verbatimLongitude: 28°21'41.16"E; **Identification:** identificationID: esoguent-th-ıd-176; **Event:** eventDate: 05/22/2002
- **Type status:** Other material. **Occurrence:** sex: 4 males pharate adult, 1 female pharate adult; lifeStage: 9 pupae, 21 larvae; **Taxon:** scientificName: Simulium (Wilhelmia) pseudequinum Seguy, 1921; kingdom: Animalia; phylum: Arthropoda; class: Insecta; order: Diptera; family: Simuliidae; genus: Simulium; subgenus: Wilhelmia; scientificNameAuthorship: Seguy, 1921; **Location:** continent: Europe; country: Turkey; stateProvince: İstanbul; county: Çatalca; locationRemarks: 13; verbatimLatitude: 41°18'19.33"N; verbatimLongitude: 28°26'3.30"E; **Identification:** identificationID: esoguent-th-ıd-177; **Event:** eventDate: 05/23/2002
- **Type status:** Other material. **Occurrence:** sex: 1 male pharate adult; lifeStage: 3 pupae, 2 larvae; **Taxon:** scientificName: Simulium (Wilhelmia) pseudequinum Seguy, 1921; kingdom: Animalia; phylum: Arthropoda; class: Insecta; order: Diptera; family: Simuliidae; genus: Simulium; subgenus: Wilhelmia; scientificNameAuthorship: Seguy, 1921; **Location:** continent: Europe; country: Turkey; stateProvince: İstanbul; county: Çatalca; locationRemarks: 14; verbatimLatitude: 41°20'19.18"N; verbatimLongitude: 28°25'28.91"E; **Identification:** identificationID: esoguent-th-ıd-178; **Event:** eventDate: 05/23/2002
- **Type status:** Other material. **Occurrence:** sex: 1 male pharate adult; lifeStage: 2 pupae; **Taxon:** scientificName: Simulium (Wilhelmia) pseudequinum Seguy, 1921; kingdom: Animalia; phylum: Arthropoda; class: Insecta; order: Diptera; family: Simuliidae; genus: Simulium; subgenus: Wilhelmia; scientificNameAuthorship: Seguy, 1921; **Location:** continent: Europe; country: Turkey; stateProvince: İstanbul; county: Çatalca; locationRemarks: 15; verbatimLatitude: 41°21'23.18"N; verbatimLongitude: 28°23'55.26"E; **Identification:** identificationID: esoguent-th-ıd-179; **Event:** eventDate: 05/23/2002
- **Type status:** Other material. **Occurrence:** sex: 1 male, 2 male pharate adult; lifeStage: 6 pupae, 5 larvae; **Taxon:** scientificName: Simulium (Wilhelmia) pseudequinum Seguy, 1921; kingdom: Animalia; phylum: Arthropoda; class: Insecta; order: Diptera; family: Simuliidae; genus: Simulium; subgenus: Wilhelmia; scientificNameAuthorship: Seguy, 1921; **Location:** continent: Europe; country: Turkey; stateProvince: İstanbul; county: Çatalca; locationRemarks: 17; verbatimLatitude: 41°23'54.45"N; verbatimLongitude: 28°19'8.26"E; **Identification:** identificationID: esoguent-th-ıd-180; **Event:** eventDate: 05/23/2002
- **Type status:** Other material. **Occurrence:** sex: 1 male pharate adult, 2 females pharate adult; lifeStage: 8 pupae, 4 larvae; **Taxon:** scientificName: Simulium (Wilhelmia) pseudequinum Seguy, 1921; kingdom: Animalia; phylum: Arthropoda; class: Insecta; order: Diptera; family: Simuliidae; genus: Simulium; subgenus: Wilhelmia; scientificNameAuthorship: Seguy, 1921; **Location:** continent: Europe; country: Turkey; stateProvince: İstanbul; county: Çatalca; locationRemarks: 20; verbatimLatitude: 41°24'2.09"N; verbatimLongitude: 28°10'3.80"E; **Identification:** identificationID: esoguent-th-ıd-181; **Event:** eventDate: 07/09/2003
- **Type status:** Other material. **Occurrence:** lifeStage: 2 pupae, 4 larvae / 1 pupae; **Taxon:** scientificName: Simulium (Wilhelmia) pseudequinum Seguy, 1921; kingdom: Animalia; phylum: Arthropoda; class: Insecta; order: Diptera; family: Simuliidae; genus: Simulium; subgenus: Wilhelmia; scientificNameAuthorship: Seguy, 1921; **Location:** continent: Europe; country: Turkey; stateProvince: Tekirdağ; county: Saray; locationRemarks: 21; verbatimLatitude: 41°26'19.63"N; verbatimLongitude: 28°3'45.95"E; **Identification:** identificationID: esoguent-th-ıd-182; **Event:** eventDate: 24.05.2002 / 09.07.2003
- **Type status:** Other material. **Occurrence:** sex: 1 male pharate adult; lifeStage: 9 pupae, 9 larvae; **Taxon:** scientificName: Simulium (Wilhelmia) pseudequinum Seguy, 1921; kingdom: Animalia; phylum: Arthropoda; class: Insecta; order: Diptera; family: Simuliidae; genus: Simulium; subgenus: Wilhelmia; scientificNameAuthorship: Seguy, 1921; **Location:** continent: Europe; country: Turkey; stateProvince: Tekirdağ; county: Saray; locationRemarks: 22; verbatimLatitude: 41°26'35.31"N; verbatimLongitude: 28°1'25.37"E; **Identification:** identificationID: esoguent-th-ıd-183; **Event:** eventDate: 24.05.2002 / 09.07.2003
- **Type status:** Other material. **Occurrence:** sex: 3 males pharate adult; lifeStage: 3 pupae, 12 larvae / 3 pupae; **Taxon:** scientificName: Simulium (Wilhelmia) pseudequinum Seguy, 1921; kingdom: Animalia; phylum: Arthropoda; class: Insecta; order: Diptera; family: Simuliidae; genus: Simulium; subgenus: Wilhelmia; scientificNameAuthorship: Seguy, 1921; **Location:** continent: Europe; country: Turkey; stateProvince: Tekirdağ; county: Saray; locationRemarks: 23; verbatimLatitude: 41°28'49.33"N; verbatimLongitude: 27°57'11.84"E; **Identification:** identificationID: esoguent-th-ıd-184; **Event:** eventDate: 24.05.2002 / 09.07.2003
- **Type status:** Other material. **Occurrence:** sex: 2 males pharate adult, 1 female pharate adult; lifeStage: 9 larvae; **Taxon:** scientificName: Simulium (Wilhelmia) pseudequinum Seguy, 1921; kingdom: Animalia; phylum: Arthropoda; class: Insecta; order: Diptera; family: Simuliidae; genus: Simulium; subgenus: Wilhelmia; scientificNameAuthorship: Seguy, 1921; **Location:** continent: Europe; country: Turkey; stateProvince: Tekirdağ; county: Saray; locationRemarks: 24; verbatimLatitude: 41°32'57.72"N; verbatimLongitude: 28°2'32.53"E; **Identification:** identificationID: esoguent-th-ıd-185; **Event:** eventDate: 05/24/2002
- **Type status:** Other material. **Occurrence:** sex: 1 male, 1 female, 1 male pharate adult; lifeStage: 9 pupae, 4 larvae; **Taxon:** scientificName: Simulium (Wilhelmia) pseudequinum Seguy, 1921; kingdom: Animalia; phylum: Arthropoda; class: Insecta; order: Diptera; family: Simuliidae; genus: Simulium; subgenus: Wilhelmia; scientificNameAuthorship: Seguy, 1921; **Location:** continent: Europe; country: Turkey; stateProvince: Tekirdağ; county: Saray; locationRemarks: 25; verbatimLatitude: 41°33'36.45"N; verbatimLongitude: 28°3'50.97"E; **Identification:** identificationID: esoguent-th-ıd-186; **Event:** eventDate: 05/24/2002
- **Type status:** Other material. **Occurrence:** sex: 2 males pharate adult, 1 female pharate adult; lifeStage: 6 pupae; **Taxon:** scientificName: Simulium (Wilhelmia) pseudequinum Seguy, 1921; kingdom: Animalia; phylum: Arthropoda; class: Insecta; order: Diptera; family: Simuliidae; genus: Simulium; subgenus: Wilhelmia; scientificNameAuthorship: Seguy, 1921; **Location:** continent: Europe; country: Turkey; stateProvince: Kırklareli; county: Kıyıköy; locationRemarks: 27; verbatimLatitude: 41°37'33.39"N; verbatimLongitude: 27°53'35.61"E; **Identification:** identificationID: esoguent-th-ıd-187; **Event:** eventDate: 05/24/2002
- **Type status:** Other material. **Occurrence:** sex: 3 males pharate adult; lifeStage: 3 pupae, 15 larvae / 2 pupae; **Taxon:** scientificName: Simulium (Wilhelmia) pseudequinum Seguy, 1921; kingdom: Animalia; phylum: Arthropoda; class: Insecta; order: Diptera; family: Simuliidae; genus: Simulium; subgenus: Wilhelmia; scientificNameAuthorship: Seguy, 1921; **Location:** continent: Europe; country: Turkey; stateProvince: Kırklareli; county: Vize; locationRemarks: 29; verbatimLatitude: 41°37'42.79"N; verbatimLongitude: 27°38'58.94"E; **Identification:** identificationID: esoguent-th-ıd-188; **Event:** eventDate: 25.05.2002 / 22.04.2005
- **Type status:** Other material. **Occurrence:** sex: 1 male pharate adult; lifeStage: 12 pupae; **Taxon:** scientificName: Simulium (Wilhelmia) pseudequinum Seguy, 1921; kingdom: Animalia; phylum: Arthropoda; class: Insecta; order: Diptera; family: Simuliidae; genus: Simulium; subgenus: Wilhelmia; scientificNameAuthorship: Seguy, 1921; **Location:** continent: Europe; country: Turkey; stateProvince: Kırklareli; county: Pınarhisar; locationRemarks: 30; verbatimLatitude: 41°41'23.10"N; verbatimLongitude: 27°37'12.44"E; **Identification:** identificationID: esoguent-th-ıd-189; **Event:** eventDate: 05/25/2002
- **Type status:** Other material. **Occurrence:** sex: 1 female, 2 males pharate adult; lifeStage: 3 pupae, 8 larvae; **Taxon:** scientificName: Simulium (Wilhelmia) pseudequinum Seguy, 1921; kingdom: Animalia; phylum: Arthropoda; class: Insecta; order: Diptera; family: Simuliidae; genus: Simulium; subgenus: Wilhelmia; scientificNameAuthorship: Seguy, 1921; **Location:** continent: Europe; country: Turkey; stateProvince: Kırklareli; county: Merkez; locationRemarks: 46; verbatimLatitude: 41°49'48.43"N; verbatimLongitude: 27°10'42.01"E; **Identification:** identificationID: esoguent-th-ıd-190; **Event:** eventDate: 05/28/2002
- **Type status:** Other material. **Occurrence:** sex: 2 males pharate adult, 1 female pharate adult; lifeStage: 9 pupae, 9 larvae; **Taxon:** scientificName: Simulium (Wilhelmia) pseudequinum Seguy, 1921; kingdom: Animalia; phylum: Arthropoda; class: Insecta; order: Diptera; family: Simuliidae; genus: Simulium; subgenus: Wilhelmia; scientificNameAuthorship: Seguy, 1921; **Location:** continent: Europe; country: Turkey; stateProvince: Kırklareli; county: Kofçaz; locationRemarks: 50; verbatimLatitude: 41°56'53.66"N; verbatimLongitude: 27°9'42.23"E; **Identification:** identificationID: esoguent-th-ıd-191; **Event:** eventDate: 05/28/2002
- **Type status:** Other material. **Occurrence:** sex: 1 male pharate adult, 1 female pharate adult; lifeStage: 5 pupae, 8 larvae; **Taxon:** scientificName: Simulium (Wilhelmia) pseudequinum Seguy, 1921; kingdom: Animalia; phylum: Arthropoda; class: Insecta; order: Diptera; family: Simuliidae; genus: Simulium; subgenus: Wilhelmia; scientificNameAuthorship: Seguy, 1921; **Location:** continent: Europe; country: Turkey; stateProvince: Edirne; county: İnece; locationRemarks: 55; verbatimLatitude: 41°40'13.00"N; verbatimLongitude: 27°4'7.90"E; **Identification:** identificationID: esoguent-th-ıd-192; **Event:** eventDate: 05/29/2002
- **Type status:** Other material. **Occurrence:** sex: 3 males pharate adult; lifeStage: 9 pupae, 9 larvae; **Taxon:** scientificName: Simulium (Wilhelmia) pseudequinum Seguy, 1921; kingdom: Animalia; phylum: Arthropoda; class: Insecta; order: Diptera; family: Simuliidae; genus: Simulium; subgenus: Wilhelmia; scientificNameAuthorship: Seguy, 1921; **Location:** continent: Europe; country: Turkey; stateProvince: Edirne; county: Merkez; locationRemarks: 58; verbatimLatitude: 41°46'33.77"N; verbatimLongitude: 26°40'36.82"E; **Identification:** identificationID: esoguent-th-ıd-193; **Event:** eventDate: 05/29/2002
- **Type status:** Other material. **Occurrence:** sex: 1 male pharate adult, 3 females pharate adult; lifeStage: 7 pupae; **Taxon:** scientificName: Simulium (Wilhelmia) pseudequinum Seguy, 1921; kingdom: Animalia; phylum: Arthropoda; class: Insecta; order: Diptera; family: Simuliidae; genus: Simulium; subgenus: Wilhelmia; scientificNameAuthorship: Seguy, 1921; **Location:** continent: Europe; country: Turkey; stateProvince: Edirne; county: Merkez; locationRemarks: 61; verbatimLatitude: 41°43'25.74"N; verbatimLongitude: 26°33'38.19"E; **Identification:** identificationID: esoguent-th-ıd-194; **Event:** eventDate: 05/30/2002
- **Type status:** Other material. **Occurrence:** sex: 1 male, 1 male pharate adult; lifeStage: 12 pupae, 14 larvae; **Taxon:** scientificName: Simulium (Wilhelmia) pseudequinum Seguy, 1921; kingdom: Animalia; phylum: Arthropoda; class: Insecta; order: Diptera; family: Simuliidae; genus: Simulium; subgenus: Wilhelmia; scientificNameAuthorship: Seguy, 1921; **Location:** continent: Europe; country: Turkey; stateProvince: Edirne; county: İskender; locationRemarks: 65; verbatimLatitude: 41°35'21.77"N; verbatimLongitude: 26°40'57.69"E; **Identification:** identificationID: esoguent-th-ıd-195; **Event:** eventDate: 05/31/2002
- **Type status:** Other material. **Occurrence:** sex: 1 male pharate adult, 1 female pharate adult; lifeStage: 6 pupae, 9 larvae; **Taxon:** scientificName: Simulium (Wilhelmia) pseudequinum Seguy, 1921; kingdom: Animalia; phylum: Arthropoda; class: Insecta; order: Diptera; family: Simuliidae; genus: Simulium; subgenus: Wilhelmia; scientificNameAuthorship: Seguy, 1921; **Location:** continent: Europe; country: Turkey; stateProvince: Edirne; county: Havsa; locationRemarks: 67; verbatimLatitude: 41°22'5.91"N; verbatimLongitude: 26°40'1.43"E; **Identification:** identificationID: esoguent-th-ıd-196; **Event:** eventDate: 06/01/2002
- **Type status:** Other material. **Occurrence:** sex: 1 male pharate adult, 1 female pharate adult; lifeStage: 5 pupae, 11 larvae / 2 pupae; **Taxon:** scientificName: Simulium (Wilhelmia) pseudequinum Seguy, 1921; kingdom: Animalia; phylum: Arthropoda; class: Insecta; order: Diptera; family: Simuliidae; genus: Simulium; subgenus: Wilhelmia; scientificNameAuthorship: Seguy, 1921; **Location:** continent: Europe; country: Turkey; stateProvince: Edirne; county: Uzunköprü; locationRemarks: 69; verbatimLatitude: 41°6'21.69"N; verbatimLongitude: 26°38'11.89"E; **Identification:** identificationID: esoguent-th-ıd-197; **Event:** eventDate: 01.06.2002 / 06.07.2003
- **Type status:** Other material. **Occurrence:** sex: 2 males, 2 females, 2 males pharate adult, 2 females pharate adult; lifeStage: 4 pupae, 7 larvae; **Taxon:** scientificName: Simulium (Wilhelmia) pseudequinum Seguy, 1921; kingdom: Animalia; phylum: Arthropoda; class: Insecta; order: Diptera; family: Simuliidae; genus: Simulium; subgenus: Wilhelmia; scientificNameAuthorship: Seguy, 1921; **Location:** continent: Europe; country: Turkey; stateProvince: Edirne; county: Keşan; locationRemarks: 70; verbatimLatitude: 41°5'0.44"N; verbatimLongitude: 26°32'11.32"E; **Identification:** identificationID: esoguent-th-ıd-198; **Event:** eventDate: 06/01/2002
- **Type status:** Other material. **Occurrence:** sex: 1 male pharate adult; lifeStage: 2 pupae; **Taxon:** scientificName: Simulium (Wilhelmia) pseudequinum Seguy, 1921; kingdom: Animalia; phylum: Arthropoda; class: Insecta; order: Diptera; family: Simuliidae; genus: Simulium; subgenus: Wilhelmia; scientificNameAuthorship: Seguy, 1921; **Location:** continent: Europe; country: Turkey; stateProvince: Edirne; county: Uzunköprü; locationRemarks: 72; verbatimLatitude: 41°2'12.05"N; verbatimLongitude: 26°24'7.33"E; **Identification:** identificationID: esoguent-th-ıd-199; **Event:** eventDate: 06/01/2002
- **Type status:** Other material. **Occurrence:** lifeStage: 2 pupae; **Taxon:** scientificName: Simulium (Wilhelmia) pseudequinum Seguy, 1921; kingdom: Animalia; phylum: Arthropoda; class: Insecta; order: Diptera; family: Simuliidae; genus: Simulium; subgenus: Wilhelmia; scientificNameAuthorship: Seguy, 1921; **Location:** continent: Europe; country: Turkey; stateProvince: Edirne; county: İpsala; locationRemarks: 73; verbatimLatitude: 40°58'31.39"N; verbatimLongitude: 26°31'58.38"E; **Identification:** identificationID: esoguent-th-ıd-200; **Event:** eventDate: 06/01/2002
- **Type status:** Other material. **Occurrence:** sex: 1 male pharate adult, 2 females pharate adult; lifeStage: 6 pupae, 11 larvae; **Taxon:** scientificName: Simulium (Wilhelmia) pseudequinum Seguy, 1921; kingdom: Animalia; phylum: Arthropoda; class: Insecta; order: Diptera; family: Simuliidae; genus: Simulium; subgenus: Wilhelmia; scientificNameAuthorship: Seguy, 1921; **Location:** continent: Europe; country: Turkey; stateProvince: Edirne; county: Keşan; locationRemarks: 75; verbatimLatitude: 40°46'5.97"N; verbatimLongitude: 26°30'42.72"E; **Identification:** identificationID: esoguent-th-ıd-201; **Event:** eventDate: 06/02/2002
- **Type status:** Other material. **Occurrence:** sex: 3 males pharate adult; lifeStage: 5 pupae / 3 pupae; **Taxon:** scientificName: Simulium (Wilhelmia) pseudequinum Seguy, 1921; kingdom: Animalia; phylum: Arthropoda; class: Insecta; order: Diptera; family: Simuliidae; genus: Simulium; subgenus: Wilhelmia; scientificNameAuthorship: Seguy, 1921; **Location:** continent: Europe; country: Turkey; stateProvince: Tekirdağ; county: Şarköy; locationRemarks: 83; verbatimLatitude: 40°46'21.52"N; verbatimLongitude: 27°3'32.09"E; **Identification:** identificationID: esoguent-th-ıd-202; **Event:** eventDate: 04.06.2002 / 05.07.2003
- **Type status:** Other material. **Occurrence:** sex: 1 male pharate adult, 1 female pharate adult; lifeStage: 9 pupae, 5 larvae; **Taxon:** scientificName: Simulium (Wilhelmia) pseudequinum Seguy, 1921; kingdom: Animalia; phylum: Arthropoda; class: Insecta; order: Diptera; family: Simuliidae; genus: Simulium; subgenus: Wilhelmia; scientificNameAuthorship: Seguy, 1921; **Location:** continent: Europe; country: Turkey; stateProvince: Çanakkale; county: Gelibolu; locationRemarks: 90; verbatimLatitude: 40°26'2.76"N; verbatimLongitude: 26°33'22.85"E; **Identification:** identificationID: esoguent-th-ıd-203; **Event:** eventDate: 06/05/2002
- **Type status:** Other material. **Occurrence:** sex: 1 male pharate adult; lifeStage: 4 pupae, 7 larvae; **Taxon:** scientificName: Simulium (Wilhelmia) pseudequinum Seguy, 1921; kingdom: Animalia; phylum: Arthropoda; class: Insecta; order: Diptera; family: Simuliidae; genus: Simulium; subgenus: Wilhelmia; scientificNameAuthorship: Seguy, 1921; **Location:** continent: Europe; country: Turkey; stateProvince: Tekirdağ; county: Malkara; locationRemarks: 93; verbatimLatitude: 40°56'45.42"N; verbatimLongitude: 26°53'8.36"E; **Identification:** identificationID: esoguent-th-ıd-204; **Event:** eventDate: 06/06/2002
- **Type status:** Other material. **Occurrence:** sex: 2 males pharate adult; lifeStage: 6 pupae, 9 larvae; **Taxon:** scientificName: Simulium (Wilhelmia) pseudequinum Seguy, 1921; kingdom: Animalia; phylum: Arthropoda; class: Insecta; order: Diptera; family: Simuliidae; genus: Simulium; subgenus: Wilhelmia; scientificNameAuthorship: Seguy, 1921; **Location:** continent: Europe; country: Turkey; stateProvince: Tekirdağ; county: Malkara; locationRemarks: 94; verbatimLatitude: 40°59'44.99"N; verbatimLongitude: 26°45'42.54"E; **Identification:** identificationID: esoguent-th-ıd-205; **Event:** eventDate: 06/06/2002
- **Type status:** Other material. **Occurrence:** sex: 1 male pharate adult, 3 females pharate adult; lifeStage: 6 pupae, 7 larvae; **Taxon:** scientificName: Simulium (Wilhelmia) pseudequinum Seguy, 1921; kingdom: Animalia; phylum: Arthropoda; class: Insecta; order: Diptera; family: Simuliidae; genus: Simulium; subgenus: Wilhelmia; scientificNameAuthorship: Seguy, 1921; **Location:** continent: Europe; country: Turkey; stateProvince: Tekirdağ; county: Malkara; locationRemarks: 97; verbatimLatitude: 41°0'48.93"N; verbatimLongitude: 26°56'39.41"E; **Identification:** identificationID: esoguent-th-ıd-206; **Event:** eventDate: 06/06/2002
- **Type status:** Other material. **Occurrence:** sex: 1 male pharate adult; lifeStage: 4 pupae, 6 larvae; **Taxon:** scientificName: Simulium (Wilhelmia) pseudequinum Seguy, 1921; kingdom: Animalia; phylum: Arthropoda; class: Insecta; order: Diptera; family: Simuliidae; genus: Simulium; subgenus: Wilhelmia; scientificNameAuthorship: Seguy, 1921; **Location:** continent: Europe; country: Turkey; stateProvince: Tekirdağ; county: Hayrabolu; locationRemarks: 100; verbatimLatitude: 41°11'28.14"N; verbatimLongitude: 27°12'29.08"E; **Identification:** identificationID: esoguent-th-ıd-207; **Event:** eventDate: 06/06/2002
- **Type status:** Other material. **Occurrence:** sex: 1 male pharate adult; lifeStage: 3 pupae, 2 larvae; **Taxon:** scientificName: Simulium (Wilhelmia) pseudequinum Seguy, 1921; kingdom: Animalia; phylum: Arthropoda; class: Insecta; order: Diptera; family: Simuliidae; genus: Simulium; subgenus: Wilhelmia; scientificNameAuthorship: Seguy, 1921; **Location:** continent: Europe; country: Turkey; stateProvince: Kırklareli; county: Babaeski; locationRemarks: 101; verbatimLatitude: 41°28'3.89"N; verbatimLongitude: 27°1'12.76"E; **Identification:** identificationID: esoguent-th-ıd-208; **Event:** eventDate: 06/07/2002
- **Type status:** Other material. **Occurrence:** sex: 1 male pharate adult, 1 female pharate adult; lifeStage: 7 pupae, 8 larvae; **Taxon:** scientificName: Simulium (Wilhelmia) pseudequinum Seguy, 1921; kingdom: Animalia; phylum: Arthropoda; class: Insecta; order: Diptera; family: Simuliidae; genus: Simulium; subgenus: Wilhelmia; scientificNameAuthorship: Seguy, 1921; **Location:** continent: Europe; country: Turkey; stateProvince: Kırklareli; county: Babaeski; locationRemarks: 103; verbatimLatitude: 41°27'8.31"N; verbatimLongitude: 27°2'44.09"E; **Identification:** identificationID: esoguent-th-ıd-209; **Event:** eventDate: 06/07/2002
- **Type status:** Other material. **Occurrence:** sex: 2 males pharate adult; lifeStage: 6 pupae, 15 larvae; **Taxon:** scientificName: Simulium (Wilhelmia) pseudequinum Seguy, 1921; kingdom: Animalia; phylum: Arthropoda; class: Insecta; order: Diptera; family: Simuliidae; genus: Simulium; subgenus: Wilhelmia; scientificNameAuthorship: Seguy, 1921; **Location:** continent: Europe; country: Turkey; stateProvince: Tekirdağ; county: Kumbağ; locationRemarks: 108; verbatimLatitude: 40°47'52.99"N; verbatimLongitude: 27°21'48.85"E; **Identification:** identificationID: esoguent-th-ıd-210; **Event:** eventDate: 06/08/2002
- **Type status:** Other material. **Occurrence:** sex: 1 male pharate adult; lifeStage: 5 pupae, 12 larvae; **Taxon:** scientificName: Simulium (Wilhelmia) pseudequinum Seguy, 1921; kingdom: Animalia; phylum: Arthropoda; class: Insecta; order: Diptera; family: Simuliidae; genus: Simulium; subgenus: Wilhelmia; scientificNameAuthorship: Seguy, 1921; **Location:** continent: Europe; country: Turkey; stateProvince: Tekirdağ; county: Şarköy; locationRemarks: 109; verbatimLatitude: 40°45'1.14"N; verbatimLongitude: 27°20'0.83"E; **Identification:** identificationID: esoguent-th-ıd-211; **Event:** eventDate: 06/08/2002
- **Type status:** Other material. **Occurrence:** sex: 1 female pharate adult; lifeStage: 2 pupae; **Taxon:** scientificName: Simulium (Wilhelmia) pseudequinum Seguy, 1921; kingdom: Animalia; phylum: Arthropoda; class: Insecta; order: Diptera; family: Simuliidae; genus: Simulium; subgenus: Wilhelmia; scientificNameAuthorship: Seguy, 1921; **Location:** continent: Europe; country: Turkey; stateProvince: Tekirdağ; county: Şarköy; locationRemarks: 110; verbatimLatitude: 40°42'53.50"N; verbatimLongitude: 27°17'43.11"E; **Identification:** identificationID: esoguent-th-ıd-212; **Event:** eventDate: 06/08/2002
- **Type status:** Other material. **Occurrence:** sex: 1 male pharate adult; lifeStage: 3 pupae, 2 larvae; **Taxon:** scientificName: Simulium (Wilhelmia) pseudequinum Seguy, 1921; kingdom: Animalia; phylum: Arthropoda; class: Insecta; order: Diptera; family: Simuliidae; genus: Simulium; subgenus: Wilhelmia; scientificNameAuthorship: Seguy, 1921; **Location:** continent: Europe; country: Turkey; stateProvince: Tekirdağ; county: Kumbağ; locationRemarks: 111; verbatimLatitude: 40°52'10.71"N; verbatimLongitude: 27°24'50.36"E; **Identification:** identificationID: esoguent-th-ıd-213; **Event:** eventDate: 06/09/2002
- **Type status:** Other material. **Occurrence:** sex: 1 male pharate adult, 1 female pharate adult; lifeStage: 3 pupae; **Taxon:** scientificName: Simulium (Wilhelmia) pseudequinum Seguy, 1921; kingdom: Animalia; phylum: Arthropoda; class: Insecta; order: Diptera; family: Simuliidae; genus: Simulium; subgenus: Wilhelmia; scientificNameAuthorship: Seguy, 1921; **Location:** continent: Europe; country: Turkey; stateProvince: Tekirdağ; county: Şarköy; locationRemarks: 115; verbatimLatitude: 40°47'10.21"N; verbatimLongitude: 27°9'40.88"E; **Identification:** identificationID: esoguent-th-ıd-214; **Event:** eventDate: 06/09/2002
- **Type status:** Other material. **Occurrence:** sex: 1 male pharate adult; lifeStage: 11 pupae; **Taxon:** scientificName: Simulium (Wilhelmia) pseudequinum Seguy, 1921; kingdom: Animalia; phylum: Arthropoda; class: Insecta; order: Diptera; family: Simuliidae; genus: Simulium; subgenus: Wilhelmia; scientificNameAuthorship: Seguy, 1921; **Location:** continent: Europe; country: Turkey; stateProvince: İstanbul; county: Çatalca; locationRemarks: 125; verbatimLatitude: 41°22'32.22"N; verbatimLongitude: 28°18'17.39"E; **Identification:** identificationID: esoguent-th-ıd-215; **Event:** eventDate: 07/09/2003
- **Type status:** Other material. **Occurrence:** lifeStage: 2 pupae; **Taxon:** scientificName: Simulium (Wilhelmia) pseudequinum Seguy, 1921; kingdom: Animalia; phylum: Arthropoda; class: Insecta; order: Diptera; family: Simuliidae; genus: Simulium; subgenus: Wilhelmia; scientificNameAuthorship: Seguy, 1921; **Location:** continent: Europe; country: Turkey; stateProvince: Tekirdağ; county: Çorlu; locationRemarks: 129; verbatimLatitude: 41°17'36.96"N; verbatimLongitude: 27°46'11.22"E; **Identification:** identificationID: esoguent-th-ıd-216; **Event:** eventDate: 05/24/2006
- **Type status:** Other material. **Occurrence:** lifeStage: 4 pupae; **Taxon:** scientificName: Simulium (Wilhelmia) pseudequinum Seguy, 1921; kingdom: Animalia; phylum: Arthropoda; class: Insecta; order: Diptera; family: Simuliidae; genus: Simulium; subgenus: Wilhelmia; scientificNameAuthorship: Seguy, 1921; **Location:** continent: Europe; country: Turkey; stateProvince: Kırklareli; county: Lüleburgaz; locationRemarks: 132; verbatimLatitude: 41°29'20.45"N; verbatimLongitude: 27°23'52.49"E; **Identification:** identificationID: esoguent-th-ıd-217; **Event:** eventDate: 05/26/2006

#### Notes

*Simulium pseudequinum* is one of the most common black fly species in the Palearctic Region, from the Canary Islands to China. It is abundantly known from all Mediterranean countries except Egypt (Crosskey and Malicky 2001). In Anatolia, it has a wide distribution and has been reported by many authors [Zwick (1978) (as *mediterraneum* Puri, 1925), Kazancı and Clergue-Gazeau (1990), Sirin and Sahin (2005), Kazancı and Ertunç (2008), Gazyağcı and Aydenizöz (2011), Şirin et al. (2014)]. We encountered *S. pseudequinum* at 43 sites, the highest number for any species, and we consider it the most abundant black fly in the region. Crosskey and Malicky (2001) emphasized that in the western Mediterranean area it is reliably recognized in the pupa by the wrinkled bases of the middle group of gill tubes. This character, however, does not suffice in the eastern Mediterranean and Middle East, where *S. pseudequinum* can be confused with *S. paraequinum*, the two species having virtually identical gills. *Simulium pseudequinum* can be distinguished by the small spermatheca, resembling an unopened mushroom, and the characteristic shape of the male ventral plate, which is narrow apically with the inner margins resembling an isosceles triangle (Crosskey and Malicky 2001​). In our specimens, the spermatheca of the females and the ventral plate of the males agreed with the description for *S. pseudequinum* given above.

### Simulium (Wilhelmia) paraequinum

Puri, 1933

#### Materials

- **Type status:** Other material. **Occurrence:** sex: 5 males pharate adult, 3 females pharate adult; lifeStage: 3 pupae, 4 larvae; **Taxon:** scientificName: Simulium (Wilhelmia) paraequinum Puri, 1933; kingdom: Animalia; phylum: Arthropoda; class: Insecta; order: Diptera; family: Simuliidae; genus: Simulium; subgenus: Wilhelmia; scientificNameAuthorship: Puri, 1933; **Location:** continent: Europe; country: Turkey; stateProvince: Kırklareli; county: Üsküp; locationRemarks: 41; verbatimLatitude: 41°48'7.98"N; verbatimLongitude: 27°27'14.63"E; **Identification:** identificationID: esoguent-th-ıd-218; **Event:** eventDate: 05/27/2002

#### Notes

*Simulium paraequinum* ranges from the Balkans to Pakistan; Anatolia lies centrally in the distribution. This species was recorded first from Anatolia by Kazancı and Clergue-Gazeau (1990) near the southwestern corner of Asia Minor. Crosskey and Zwick (2007) recorded it from five sites in the southern Taurus Mountains and northwestern Anatolia. It also occurs in Greece and Bulgaria, which are boundary countries of Turkish Thrace. This species can be distinguished from *S. pseudequinum* only by the genitalia of males and females. The genitalia of our pharate males and females collected from only one locality, Site 41, fit the figure and description of *S. paraequinum* given by Crosskey and Malicky (2001)​. We observed that the length of the gill tubes of our specimens of *S. paraequinum* were longer than all of those of *S. pseudequinum*.

## Discussion

The simuliid species composition of Turkish Thrace is similar to that of Anatolia and the Balkan Countries, differing only by the presence of *Metacnephia nigra* in Turkish Thrace. The most two abundant species are respectively Simulium (Wilhelmia) pseudequinum recorded at 43 sites and Simulium (Eusimulium) petricolum from 41 sites in the region. It is known that these two species are common in Meditaranean region and can be found in different types of running waters. On the other hand, Simulium (Simulium) noelleri and Simulium (Simulium) bukovskii are reported from only one site in the region depending on their special habitat preferences.

Our survey was based only on morphotaxonomic methods and did not reveal cryptic species in complexes such as the *S. cryophilum* complex. More comprehensive taxonomic surveys, including cytotaxonomical and molecular techniques, are required to obtain further information about the faunal structure of the family in the region.

Our results show that blood-feeding species, like *Simulium erythrocephalum*, *S. reptans* and *S. bezzii*, live also in the region. Therefore, monitoring programmes for pest populations of black flies in the region are needed to ensure the public health of citizens (e.g., about 13 million people in Istanbul) and protection of livestock, especially with regard to dam-construction projects, excessive pollution of freshwater systems caused by agricultural and industrial activities, and the effects of global warming.

## Supplementary Material

Supplementary material 1 Collection sites for black flies from Turkish Thrace, 2002–2006. Data type: table
File: oo_38635.txt U.Sirin

Supplementary material 2 taxa data Data type: taxa
File: oo_39075.xls Umit Sirin

XML Treatment for Prosimulium rachiliense

XML Treatment for Metacnephia nigra

XML Treatment for Simulium (Boophthora) erythrocephalum

XML Treatment for Simulium (Eusimulium) petricolum

XML Treatment for Simulium (Eusimulium) velutinum

XML Treatment for Simulium (Nevermannia) cryophilum complex

XML Treatment for Simulium (Simulium) bezzii

XML Treatment for Simulium (Simulium) bukovskii

XML Treatment for Simulium (Simulium) noelleri

XML Treatment for Simulium (Simulium) kiritshenkoi

XML Treatment for Simulium (Simulium) reptans

XML Treatment for Simulium (Simulium) variegatum

XML Treatment for Simulium (Simulium) argyreatum

XML Treatment for Simulium (Trichodagmia) auricoma

XML Treatment for Simulium (Wilhelmia) equinum

XML Treatment for Simulium (Wilhelmia) balcanicum

XML Treatment for Simulium (Wilhelmia) pseudequinum

XML Treatment for Simulium (Wilhelmia) paraequinum
